# Analysis of the inhibiting activity of reversion-inducing cysteine-rich protein with Kazal motifs (RECK) on matrix metalloproteinases

**DOI:** 10.1038/s41598-020-63338-4

**Published:** 2020-04-14

**Authors:** Soraia R. Mendes, Laura del Amo-Maestro, Laura Marino-Puertas, Iñaki de Diego, Theodoros Goulas, F. Xavier Gomis-Rüth

**Affiliations:** 1 Proteolysis Laboratory, Department of Structural Biology, Molecular Biology Institute of Barcelona, Higher Scientific Research Council (CSIC), Barcelona Science Park, Helix Building, Baldiri Reixac, 15-21, 08028 Barcelona, Catalonia Spain; 2 Present Address: ALBA Synchrotron Light Source, Carrer de la Llum, 2-26, 08290 Cerdanyola del Vallés, Catalonia Spain

**Keywords:** Biochemistry, Biological techniques, Molecular biology

## Abstract

Matrix metalloproteinases (MMPs) occur in 23 human paralogues with key functions in physiology, and their activity is controlled by protein inhibitors. Reversion-inducing cysteine-rich protein with Kazal motifs (RECK), which is essential for embryogenesis and tumour suppression, has been reported to inhibit MMPs. Here, we developed eukaryotic and bacterial expression systems for different RECK variants and analysed their inhibitory capacity against representative MMPs *in vitro*. We could not detect any significant inhibition. Instead, we found that partially purified RECK from the conditioned medium of transfected Expi293F cells but not that of ExpiCHO-S or *Drosophila* Schneider cells contained a contaminant with proteolytic activity. The contaminant was removed through treatment with a small-molecule serine peptidase inhibitor and additional chromatographic purification. A tantamount contaminant was further detected in an equivalent expression system of the N-terminal fragment of the proteoglycan testican 3, but not in those of two other proteins. These results indicate that previous reports of inhibitory activity of recombinant RECK on MMPs, which were performed with partially purified samples, were probably masked by a coeluting contaminant present in the supernatant of HEK293-derived cells. Thus, RECK is probably not a direct inhibitor of MMP catalytic activity but may still regulate MMPs through other mechanisms.

## Introduction

Proteolysis is a post-translational modification of proteins and peptides that is essential for all physiological pathways. It is exerted by peptidases, among which metallopeptidases (MPs) are one of several chemical classes and consist of various clans and families^1^. The matrix metalloproteinases (MMPs; 23 paralogues in humans), as well as the ADAMs/adamalysins (19 in humans) and the more distantly related ADAMTSs (19 in humans) are among the most studied MPs because of their enormous relevance for human health and disease^2–8^. Collectively, they carry out functions as broad degraders during the digestion of intake proteins, turnover of extracellular-matrix components for tissue remodelling and developmental processes, and clearance of obsolete or malfunctioning polypeptides. Moreover, they are fine regulators of shedding, maturation and inactivation of other proteins through limited proteolysis of one or a few peptide bonds^9^. Peptide-bond scission is normally irreparable under physiological conditions, so MPs must be fastidiously controlled to avoid aberrant cleavage that would cause dysfunction and pathology. This regulation is physiologically carried out for MMPs in humans by four tissue inhibitors of metalloproteinases and the broad-spectrum pan-peptidase inhibitor α_2_-macroglobulin^3,10–12^. Another reported inhibitor is the protein RECK^13–17^.

**Figure 1:**
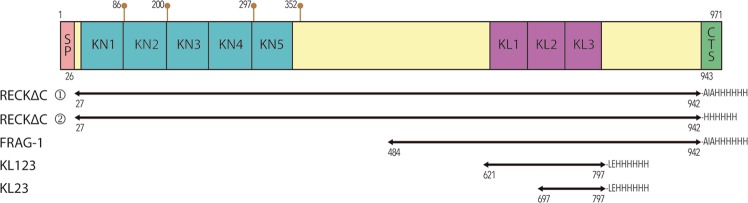
Overview of RECK constructs. Scheme depicting the domain structure of human RECK. SP, signal peptide; KN1-KN5, cysteine knot regions; KL1-KL3, Kazal-like domains; and CTS, C-terminal segment. The distinct constructs that were studied in this work are shown and labelled. ①, RECKΔC produced in Expi or Expi-CHO cells. ②, RECKΔC produced in S2 cells. Brown lollipops pinpoint glycosylation sites according to^22^.

RECK, an acronym for reversion-inducing cysteine-rich protein with Kazal motifs, is encoded by a gene that suppresses the transformed phenotype caused by *ras* oncogenes^13,18^. The 971-residue molecule is a membrane-anchored glycoprotein of ~125 kDa, which contains an N-terminal signal peptide for secretion, a region spanning five cystine knots (KNs; KN1-KN5), a region with three repeats similar to Kazal inhibitors of serine endopeptidases (KLs; KL1-KL3)^19,20^, and a C-terminal segment (CTS; residues A^943^-N^971^; for numbering, see UniProt database entry [UP] O95980) (Fig. 1). The CTS is removed during maturation, which leads to binding of RECK to the plasma membrane through a glycosylphosphatidylinositol anchor attached to S^942^ ^13,21^. In addition, *N*-linked glycosylations have been determined at residues N^200^, N^86^, N^297^ and N^352^, and the latter three are essential for function^22^.

Physiologically, RECK is critical because knockout mice die during embryonic development with severe tissue, vascular, and neuronal defects^14,23^. It is highly expressed in most normal tissues and non-transformed cells, and is probably involved in myogenesis, chondrogenesis, patterning during embryogenesis, and the establishment of the neuromuscular junction^13,17,24^. It also participates in Notch-dependent neurogenesis, Wnt signalling and brain angiogenesis^25–27^. Moreover, it has been implicated in tumour processes including growth, angiogenesis, invasion, metastasis and relapse^14,28,29^. It is downregulated with poor prognosis for disease outcome in pancreatic, oral, breast, prostate and non-small cell lung cancers, as well as in osteosarcoma^30–35^. Consistently, restored expression of RECK in tumour cells suppresses angiogenesis, invasion and metastasis in animal models, and the level of residual RECK expression in tumour tissues correlates with better prognosis^17^. All these findings suggest that RECK has a potential therapeutic value as a tumour suppressor for the treatment of malignant conditions^21^.

At the molecular level, RECK binds ADAMTS-10, which is involved in connective-tissue development^36^, and impairs modulation of Notch signalling during neurogenesis mediated by ADAM-10^23^. RECK without the CTS (RECKΔC) has further been described to prevent cleavage by ADAM-10 of a fragment of protein Delta1 and of a synthetic substrate with an apparent inhibition constant (*K*_i_) of <15 nM^23^. Moreover, RECKΔC has been claimed to directly inhibit cleavage of fluorogenic peptide substrates by MMP-2, MMP-9 and MMP-14 with associated *K*_i_ values of 20–80 nM^13–15,17^, and of plasma fibronectin by both MMP-2 and MMP-7, the latter with a *K*_i_ of ~41 nM^17^. In a report by another group^16^, tagged RECKΔC and shorter constructs spanning the two C-terminal Kazal-like motifs (P^676^-V^799^), the cysteine knots (residues L^285^-R^368^) and all three Kazal-like motifs (residues A^605^-V^799^), respectively, were assayed for inhibition of MMP-9 with fluorescein-conjugated gelatine as substrate. The authors have reported that the two former constructs but not the two latter significantly inhibit gelatine cleavage^16^. Based on all these reports, RECK has been included as an MMP inhibitor in the MEROPS database under family I1, which groups the Kazal family of inhibitors of serine endopeptidase families S1 and S8^19,20,37^.

Prompted by these results, we embarked on a long-term project to characterize the structure and function of RECK. To this aim, we here produced several constructs of the protein with the highest purity as a requisite for molecular and biophysical studies, and we assayed their inhibitory capacity against MMPs *in vitro*.

## Materials and Methods

### Expression vectors

Plasmid pBS-hRECK (for details on constructs, plasmids, vectors and primers, see Table 1) encoding full-length human RECK cDNA in the pBlueScript vector was kindly provided by Makoto Noda, Kyoto (Japan). Constructs encoding fragments RECK∆C (plasmid pS6-RECKΔC; residues G^27^-S^942^), KL123 (pCri9a-KL123; S^621^-S^797^) and KL23 (pCri9a-KL23; T^697^-S^797^) (Fig. 1), as well as the coding sequence for residues A^22^-Q^313^ (UP Q9BQ16) of the N-terminal region of human testican 3 (N-TES; plasmid pS6-NTES) in a synthetic gene (from GenScript) were amplified with primers that introduced sites for directional cloning. For bacterial expression, plasmid pCri9a^38^, which adds a C-terminal hexahistidine (His_6_)-tag, was used for insertion between the *Nco*I and *Xho*I restriction sites. For expression in mammalian cells, vector pCMV-Sport 6 (Thermo Scientific) with the original signal peptide (for RECK∆C) or with the mouse immunoglobulin κ leader sequence (for N-TES) was used to insert genes between *Sma*I/*Asi*SI and *Bst*EII/*Asi*SI restriction sites, respectively. For expression of RECKΔC in insect cells, the RECK gene was inserted into vector pIEx (Novagen) by restriction-free cloning in frame with the signal peptide of adipokinetic hormone as previously described^39^ to yield plasmid pIE-RECKΔC. Primers and DNA-modifying enzymes for polymerase chain reaction (PCR) steps were purchased from Sigma-Aldrich and Thermo Scientific, respectively. PCR was performed with Phusion High Fidelity DNA polymerase (Thermo Scientific) according to the manufacturer’s instructions with an extra optimization step by thermal gradient after each reaction. DNA was purified with the OMEGA Biotek Purification Kit (Omega) or GeneJET Plasmid MaxiPrep Kit (Thermo Scientific) according to the manufacturer’s instructions, and all constructs were verified by DNA sequencing. Plasmid pET3a-MT1∆C (see Table 1) encoding the pro- and catalytic domains of MMP-14 (S^24^-G^284^; UP P50281) *plus* an extra N-terminal methionine^40^ was kindly provided by Yoshifumi Itoh, Oxford (UK).

**Table 1:** Constructs, primers, plasmids and proteins.

Plasmid name	Protein	Parental vector(s)	Forward-primer*	Reverse-primer*	Protein sequence **	Tag***
pS6-RECK∆C	RECK∆C	pBS-hRECK pCMV-Sport6	ATGCCCCGGGATGGCGACCGTCCGG	GCATGCGATCGCCGATGGCACACTGCTG	G^27^-S^942^ + **AIA** + **H**_**6**_	His_6_
pIE-RECK∆C	RECK∆C	pBS-hRECK pIEx	TCATCGCTTTCGTCATCATCGCTGGGCCTGGCTCCGGGCAGTGCGGGTG	AAACTCAATGGTGATGGTGATGATGCGATGGCACACTGCTGGAGACCTGT	G^27^- S^942^ + **H**_**6**_	His_6_
pS6-NTES	N-TES	Synthetic DNA pCMV-Sport6	ATGCGGTGACCTAGCTGCCGCGGCGGT	GCATGCGATCGCTTGCTGTCTCTGGAAGCA	**L** + A^22^-Q^313^ + **AIA** + **H**_**6**_	His_6_
pCri9a-KL23	KL23	pS6-RECK∆C pCri9a	ATGCCCATGGTAACGACTTTTGATAA	GCATCTCGAGGCTGTGCTCTGAGAGG	**V** + T^697^-S^797^ + **LE** + **H**_**6**_	His_6_
pCri9a-KL123	KL123	pS6-RECK∆C pCri9a	ATGCCCATGGTATCAGAAGATGACCG	GCATCTCGAGGCTGTGCTCTGAGAGG	**V** + S^621^-S^797^ + **LE** + **H**_**6**_	His_6_
pET3a-MT1∆C	MMP-14 CD	pET3a	—	—	**M** + S^24^-G^284^	None

All constructs are for extracellular expression of the respective proteins.

*Restriction-site sequences and overhangs for restriction-free cloning are underlined.

**Peptide sequence of the expressed protein. Amino acids derived from the construct are in bold. See also Fig. 1.

***Tag fused to the carboxy-terminus.

### Eukaryotic cell culture and transient transfection

*Drosophila melanogaster* embryonic Schneider cells (S2; Gibco) adapted to suspension, as well as the HEK293-derived Expi293F cells (Expi; Gibco) and ExpiCHO-S derived from Chinese hamster ovary cells (Expi-CHO; Gibco), were maintained in Sf-900 II SFM and FreeStyle F17 expression medium (Gibco) for insect and mammalian cells, respectively. Both media were supplemented with 0.5 µg/mL amphotericin B (Gibco), 100 units/mL penicillin and 100 μg/mL streptomycin (Sigma). Additionally, FreeStyle F17 medium was supplemented with 8 mM L-glutamine and 0.2% Pluronic F-68 (Gibco).

Expi and Expi-CHO cells were grown to a density of 3–5 × 10^6^ cells/mL and 4–6 × 10^6^ cells/mL, respectively, and subcultured every 3–4 days by dilution to 0.3–0.5 × 10^6^ cells/mL and 0.2–0.3 × 10^6^ cells/mL, respectively. To this aim, they were incubated at 37 °C in a Multitron Cell Shaker Incubator (Infors HT) at 150 rpm in humidified atmosphere with 8% CO_2_. Cells were then subcultured to 0.7 × 10^6^ cells/mL and transfected after 24 h at a cell density of 1 × 10^6^ cells/mL with a dropwise added mixture of 1 mg of vector DNA (see Table 1) and 3 mg of linear 25-kDa polyethyleneimine (Polysciences Europe) in 20 mL of Opti-MEM medium (Gibco) per litre of expression medium. The mixture had been previously incubated at room temperature for 15–20 min. After 3 days, the cell-culture supernatant was harvested for protein purification.

S2 cells were grown to a density of 12–16 × 10^6^ cells/mL, subcultured by dilution to 4 × 10^6^ cells/mL every 3–4 days and incubated at 28 °C in an Innova 42 Incubator Shaker (New Brunswick Scientific) under agitation at 200 rpm. Cells were subcultured to 6 × 10^6^ cells/mL and transfected after 24 h at a cell density of 12 × 10^6^ cells/mL with a dropwise added mixture of 0.6 μg of DNA (see Table 1) and 2 μg of linear 25-kDa polyethyleneimine per 10^6^ cells. The mixture had been previously incubated at room temperature for 15–30 min. Transfected cells were diluted to 4 × 10^6^ cells/mL after 1 h incubation at 28 °C under agitation at 200 rpm, and the cell-culture supernatant was harvested after 7 days for protein purification.

### Bacterial expression

Plasmids pCri9a-KL123 and pCri9a-KL23 were transformed into competent Lemo21 (DE3) *Escherichia coli* cells (New England Biolabs) and plated on Luria-Bertani (LB) plates. Fifty millilitres of lysogeny broth was inoculated with a single bacterial colony and incubated overnight at 37 °C under stirring at 220 rpm. Five millilitres of this preinoculum was used to inoculate 500 mL of lysogeny broth, and cells were left to grow at 37 °C until OD_600_ ≈ 0.7. Subsequently, cultures were cooled to 20 °C and protein expression was induced with 0.4 mM isopropyl-β-D-1-thiogalactopyranoside (IPTG; Duchefa) for 18–20 h. LB plates and lysogeny broth were supplemented with 50 μg/mL kanamycin (Fisher Bioreagents) and 34 μg/mL chloramphenicol (Fluka).

For the expression of MMP-14 catalytic domain (CD), *E. coli* BL21 (DE3) cells (Sigma) were transformed with plasmid pET3a-MT1∆C. One hundred millilitres of lysogeny broth was inoculated with a single colony and incubated overnight at 28 °C under stirring at 200 rpm. Ten millilitres of this preinoculum was used to inoculate 500 mL of lysogeny broth, and cells were left to grow at 37 °C until OD_600_ ≈ 0.6. Cells were then induced with 0.5 mM IPTG and kept for 5 h at 37 °C. LB plates and lysogeny broth were supplemented with 100 μg/mL ampicillin (Apollo Scientific).

### Protein purification

For purification of RECK∆C from Expi cells, cell-culture supernatant was cleared at 4 °C by centrifugation at 3,500 × *g* for 30 min, filter-sterilized and concentrated 20-fold with a VivaFlow 200 Cross Flow Cassette device with a Hydrosart membrane of 30-kDa cutoff (Sartorius). Concentrated supernatant was then dialysed against a 75-fold volume excess of buffer 20 mM Tris·HCl pH 7.5, 150 mM sodium chloride. After addition of 20 mM imidazole to the dialysed supernatant, RECK∆C was captured by nickel-nitrilotriacetic acid (Ni-NTA) affinity chromatography (AC) in a HisTrap HP column (GE Healthcare) previously washed with buffer A (50 mM Tris·HCl pH 7.5, 1 M sodium chloride, 500 mM imidazole) and equilibrated with buffer A without imidazole. The protein was washed and eluted with a step gradient of imidazole (2%, 4%, 12% and 60% of buffer A). The presence of a proteolytic impurity in fractions containing RECK∆C was assessed through incubation with 1 mg/mL fibrinogen from human plasma (Sigma) in buffer B (50 mM Tris·HCl pH 7.5, 150 mM sodium chloride, 5 mM calcium chloride, 50 µM zinc chloride) overnight at 37 °C. To remove this contaminant and obtain highly pure RECK∆C, the protein was incubated for 1 h at room temperature with 1 mg/mL 4-[2-aminoethyl]benzenesulfonyl fluoride (AEBSF, commercial name Pefabloc, Sigma) and further purified by size exclusion chromatography (SEC) in a Superdex200 (GE Healthcare Life Sciences) column equilibrated with buffer A without imidazole.

For the production and purification of RECK construct FRAG-1 (see Fig. 1), highly purified RECK∆C was incubated with 20-fold molar excess of MMP-14 CD in buffer B overnight at 37 °C. Cleavage fragments were purified by SEC in a Superdex75 (GE Healthcare Life Sciences) column equilibrated with buffer C (50 mM Tris·HCl pH 7.5, 150 mM sodium chloride). Presence of proteolytic activity in the fractions containing FRAG-1 was assessed as above with fibrinogen.

For purification of RECK∆C from S2 or Expi-CHO cells, cleared cell culture supernatant was dialysed against a 17-fold volume excess of buffer 20 mM Tris·HCl pH 7.4, 250 mM sodium chloride, 20 mM imidazole. RECK∆C in the supernatant was captured by AC with Ni-NTA resin (Thermo Scientific) by overnight incubation at 4 °C. It was subsequently loaded onto an open column for batch purification (Bio-Rad), and washed extensively and eluted with 4% and 60% of buffer A, respectively. The presence of proteolytic activity was assessed as above with fibrinogen. Partially purified protein was further purified by SEC in a Superdex 200 10/300(GE Healthcare) column equilibrated with buffer C (RECK∆C from S2 cells) or buffer A without imidazole (RECK∆C from Expi-CHO cells).

For N-TES purification, cleared cell culture supernatant was supplemented with 20 mM imidazole and incubated for 3–4 h with Ni-NTA resin. It was subsequently loaded onto an open column for batch AC purification (Bio-Rad), and washed extensively and eluted with 4% and 60% buffer A, respectively. Eluted fractions were pooled, desalted and concentrated, and the presence of proteolytic activity was assessed as above with fibrinogen. This activity was suppressed as described above and subsequent purification by SEC followed in a Superdex 75 10/300(GE Healthcare) column equilibrated with buffer A without imidazole.

For purification of RECK constructs KL23 and KL123, bacterial cells were harvested by centrifugation at 3,500 × *g* for 30 min at 4 °C and resuspended in cold buffer 50 mM Tris·HCl pH 7.5, 250 mM sodium chloride, 2 mM ethylenediaminetetraacetate (EDTA). Cells were lysed with a cell disrupter (Constant Systems) at a pressure of 1.35 kBar, and nonclassical inclusion bodies were recovered by centrifugation at 48,000 × *g* for 30 min at 4 °C and washed first with buffer 100 mM Tris·HCl pH 7.5, cOmplete EDTA-free (inhibitor cocktail; Roche, Sigma), 2 M urea, 2% Triton X-100, and then twice with buffer D (50 mM Tris·HCl pH 7.5, inhibitor cocktail, 2 M urea). The washed inclusion bodies were resuspended in buffer D and kept for 48 h under stirring at room temperature. Non-solubilised protein was removed by centrifugation at 48,000 × *g* for 30 min at 4 °C, and the supernatant was supplemented with 20 mM imidazole. Protein was captured by AC in a HisTrap HP column (GE Healthcare) previously washed with buffer E (50 mM Tris·HCl pH 7.5, 250 mM sodium chloride, 500 mM imidazole) and equilibrated with buffer 50 mM Tris·HCl pH 7.5, 250 mM sodium chloride, 20 mM imidazole. The RECK fragments were washed and eluted with 20 mM and 300 mM imidazole (0% and 60% of buffer E), respectively. Partially purified proteins were polished by SEC in a Superdex75 (GE Healthcare) column with buffer C.

Pure MMP-14 CD was obtained from inclusion bodies by adapting a published protocol^41^. Accordingly, bacterial cells were harvested by centrifugation at 3,500 × *g* for 30 min at 4 °C and washed with 20 mM Tris·HCl pH 8.0, 20% sucrose for 10 min at 37 °C under stirring at 220 rpm. Subsequently, cells were resuspended in buffer F (20 mM Tris·HCl pH 8.0) and kept under gentle agitation overnight at room temperature. Afterwards, first deoxycholate (Sigma) at 1.25 mg/mL and then DNase I (Roche) at 1 mg/mL were added to the lysed cells for 3 h. After a further 2 h incubation, inclusion bodies were harvested by centrifugation at 6,500 × *g* for 15 min at 4 °C and resuspended in buffer F with 0.5% Triton X-100. Inclusion bodies were then dissolved in buffer 20 mM Tris·HCl pH 8.6, 50 µM zinc chloride, 20 mM 1,4-dithio-D,L-threitol (DTT; Thermo Scientific), 8 M urea. They were further purified by ion exchange chromatography (IEC) in a 6-mL Resource Q column (GE Healthcare), previously washed with buffer G (20 mM Tris·HCl pH 8.6, 0.4 M sodium chloride, 50 µM zinc chloride, 1 mM DTT, 8 M urea) and equilibrated with buffer G without sodium chloride. A step gradient of 0%, 25%, 50% and 100% of buffer G was applied and fractions containing protein were pooled. These were then diluted to 0.2 mg/mL with buffer 50 mM Tris·HCl pH 8.6, 150 mM sodium chloride, 5 mM calcium chloride, 100 µM zinc chloride, 1 mM DTT, 6 M urea, supplemented with cystamine (20 mM) and folded in two consecutive dialysis steps at 4 °C. The first step was performed overnight against a 10-fold volume excess of buffer 50 mM Tris·HCl pH 8.6, 150 mM sodium chloride, 5 mM calcium chloride, 100 µM zinc chloride, 5 mM β-mercaptoethanol, 1 mM 2-hydroxyethyldisulfide. The second step was performed against a 10-fold volume excess of buffer B, twice for 4 hours and then overnight. This procedure caused activation of MMP-14 under removal of the pro-domain. Precipitated protein was removed by centrifugation at 48,000 × *g* for 30 min at 4 °C. Subsequently, MMP-14 CD was concentrated and further purified by SEC in a Superdex75 (GE Healthcare) column with buffer B.

Other procedures applied were similar to those of previous publications of the group, e.g.^42^. In particular, protein identities and purities were assessed by 10–14% Glycine SDS-PAGE gels stained with Coomassie Brilliant Blue, by peptide mass fingerprinting of tryptic protein digests and by N-terminal sequencing through Edman degradation. The latter two analyses were carried out at the Protein Chemistry Service and Proteomics Facilities of the Centro de Investigaciones Biológicas (Madrid, Spain). Ultrafiltration steps were performed with Vivaspin 15, Vivaspin 2 and Vivaspin 500 filter devices of 3-to-30-kDa cutoff (Sartorius Stedim Biotech). Protein concentrations were generally estimated by measuring the OD_280_ in a spectrophotometer (NanoDrop; GE Healthcare) and applying the respective theoretical extinction coefficients. Particular concentrations were also measured by the BCA Protein Assay Kit (ThermoFisher Scientific) with bovine serum albumin as a standard.

### Multi-angle laser light scattering

To determine the real molecular mass of RECKΔC, multi-angle laser light scattering (SEC-MALLS) was performed as previously reported^42^ in a Dawn Helios II apparatus (Wyatt Technologies) coupled to a SEC Superdex 200 10/300 Increase column equilibrated in buffer 20 mM Tris·HCl pH 7.4, 150 mM sodium chloride at 25 °C at the joint IBMB/IRB Crystallography Platform, Barcelona Science Park (Catalonia, Spain). ASTRA 7 software (Wyatt Technologies) was used for data processing and analysis, for which a typical dn/dc value for proteins (0.185 mL/g) was assumed. All experiments were performed in triplicate.

### Proteolytic inhibition assays

Inhibition assays with fluorogenic protein and peptide substrates were performed in a microplate fluorimeter (Infinite M200, TECAN) in 100 μL reaction volumes. Proteolytic activity of MMP-2, MMP-7 and MMP-9 (all from R&D Systems) was measured with the fluorescence-based EnzCheck Assay Kit containing DQ Gelatin (*λ*_ex_ = 490 nm and *λ*_em_ = 520 nm) as fluorescein conjugate (Invitrogen) at 12.5 μg/mL. Peptidolytic activity of MMP-14 CD was measured with the fluorogenic substrate FS-6 (Mca-K-P-L-G-L-Dnp-Dpa-A-R-NH_2_; *λ*_ex_ = 325 nm and *λ*_em_ = 400 nm; Sigma) at 5 µM. Reactions were carried out at 37 °C in buffer H (50 mM Tris·HCl pH 7.5, 150 mM sodium chloride, 10 mM calcium chloride, 50 µM zinc chloride, 0.05% Brij-35) except for MMP-2 and MMP-7, for which buffer H supplemented with 1 mM 4-aminophenylmercuric acetate (APMA, Sigma) was used to activate the respective zymogens by incubation for 1 h at 37 °C. Inhibition was measured after preincubation of a 2-, 5-, 10-, 50- and 100-fold molar excess of tester proteins (KL23, KL123, RECK∆C, FRAG-1 and N-TES) with MMP-2 (0.35 ng), MMP-7 (9.8 ng), MMP-9 (2.5 ng) or MMP-14 CD (50 ng) for 1 h at 37 °C. Substrates were added to the reaction mixture and the residual proteolytic activity was measured over a timespan of 3 h. Relative activities of MMP-2, MMP-7 and MMP-9 against fluorogenic protein substrates were determined from the slope of a fluorescence vs. time curve. In contrast, fluorogenic peptides were cleaved too fast for proper slope determination, so the relative activity of MMP-14 in front of FS-6 was determined from the absolute fluorescence values measured between 40 and 50 min after reaction start. Control activity of KL23, KL123, RECKΔC, FRAG-1, N-TES and BSA was measured between 110 and 120 min after reaction start. Bovine serum albumin (BSA; Sigma) at 100-fold molar excess and o-phenanthroline (Fluka) at 5 mM were included as negative and positive controls for inhibition, respectively. In addition, inhibition assays against cleavage of human plasma fibronectin (pFN, MP Biomedicals) were evaluated by Western blot analysis (see below). MMP-2 or MMP-7 (at 40 nM) were incubated in buffer H *plus* 1 mM APMA for activation with pFN (4 nM) at 37 °C for 0, 1, 2, 4, 6, 8 or 18 h with or without RECK∆C (400 nM). The broad-spectrum MMP inhibitors marimastat (Sigma), EDTA (Fluka), o-phenanthroline, and batimastat (Calbiochem) were used in controls, as well as the serine peptidase inhibitors AEBSF and phenylmethanesulfonyl fluoride (PMSF; Acros Organics) *plus* the cOmplete EDTA-Free inhibitor cocktail.

### Western blot analyses

Protein samples were separated by 10% Glycine SDS-PAGE, transferred to Amersham Protran Premium NC Nitrocellulose Membranes (GE Healthcare Life Sciences) and blocked for one hour under gentle stirring at room temperature with 50 mL of blocking solution (5% BSA in phosphate buffered saline [PBS] *plus* 0.2% Tween 20 [PBS-T]; Sigma). Fibronectin was detected by overnight incubation at 4 °C with a rabbit polyclonal primary antibody (Abcam) diluted 1:5,000 in PBS-T with 1% BSA and subsequent incubation for 2 h at room temperature with an anti-rabbit HRP-conjugated secondary antibody (Sigma) diluted 1:8,000 with PBS-T. Blots were incubated with mild stripping buffer (1.5% glycine pH 2.2, 0.1% SDS, 1% Tween 20) and further washed with PBS and PBS-T under gentle agitation at room temperature. Blots were re-blocked and re-probed.

His_6_-tagged proteins were detected with the monoclonal His-HRP Conjugated Antibody (Santa Cruz Biotechnology) diluted 1:5,000 in PBS-T with 1% BSA incubated overnight at 4 °C and subsequently visualized with an enhanced chemiluminescence system (Super Signal West Pico Chemiluminescent; Pierce) according to the manufacturer’s instructions. Membranes were exposed to Hyperfilm ECL films (GE Healthcare Life Sciences).

### Miscellaneous

Structure prediction calculations through threading were performed with LOMETS^43^ and RAPTORX^44^ with standard parameters.

## Results and Discussion

### Protein preparation

**Figure 2:**
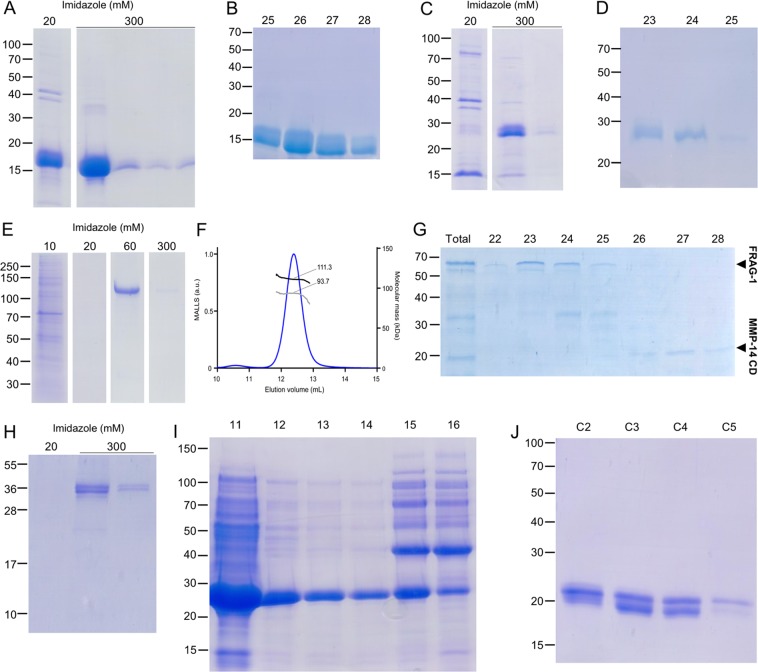
Protein purification. SDS-PAGE of AC and SEC purification steps of KL23 (**A,B**) and KL123 (**C,D**). (**E**) SDS-PAGE of stepwise AC of RECKΔC from Expi cells. (**F**) SEC-MALLS chromatogram of RECK∆C showing it has a total molecular mass of 111.3 kDa of which 93.7 kDa would correspond to protein. (**G**) SDS-PAGE of SEC fractions containing FRAG-1 (22–25) and MMP-14 CD (26–28). (**H**) SDS-PAGE of partially purified N-TES after AC purification. (**I**) SDS-PAGE of AEC purification of MMP-14 CD. (**J**) SDS-PAGE of the SEC fractions containing MMP-14 CD (C2-C5). MMP-14 CD migrates as two bands as previously observed for construct MT1Cat in^41^. Figure panels with lanes/parts from different gels/blots show white separation lines. All original gels can be found in the supplementary materials.

Inhibitory activity of RECK on MMPs *in vitro* has been reported for RECKΔC^13–15,17,23^ and a construct spanning the KL2 and KL3 domains^16^. As there were discrepancies in the boundaries of these domains^13,16^, we performed structure prediction through threading of segment V^600^-A^800^. These calculations suggested that constructs KL123 and KL23 should actually span segments V^621^-S^797^ and T^697^-S^797^, respectively (Fig. 1), which do not contain any of the glycosylation sites of the full-length protein^22^. Follistatin (Protein Data Bank [PDB] entries 2P6A, 3HH2 and 2B0U), a regulator of ligands of the transforming growth factor-β superfamily with three Kazal-like repeats^45^, and follistatin-like protein 3 (PDB 3B4V) were identified as the closest structural relatives. Both RECK constructs were produced with C-terminal His_6_-tags overnight in *E. coli* Lemo21 cells at room temperature and translocated to the periplasm, which provides an oxidizing environment for the formation of disulphide bonds and protein folding. The proteins were obtained in high yields as nonclassical inclusion bodies^46^, which were treated under non-reducing conditions with a chaotropic agent and detergent prior to purification by AC and SEC steps (Fig. 2A–D). The resulting proteins were soluble and highly pure, did not aggregate when concentrated, and migrated according to 16 kDa (KL23) and 26 kDa (KL123) in calibrated SEC (data not shown), which are consistent with monomeric species. These data suggest that the proteins were well-folded.

We also isolated RECKΔC with a C-terminal His_6_-tag (Fig. 1) from the conditioned medium of transiently transfected Expi cells by adapting a protocol developed previously for human α_2_-macroglobulin^42^. The yield after purification was 0.8 mg per litre of expression medium, and the protein was subsequently purified by AC and SEC (Fig. 2E). It had a molecular mass of 111 kDa according to SEC-MALLS (Fig. 2F), which is in good agreement with the theoretical protein mass *plus* glycosylation, and indicates that the protein is monomeric. This contrasts with other studies postulating it is a dimer^17^. We further produced RECKΔC from S2 and Expi-CHO systems but the initial yields were significantly lower (0.2 and 0.5 mg/L, respectively) and the proteins required several additional purification steps (data not shown). We next obtained N-TES by the same method from transfected Expi cells (Fig. 2H). This protein spans the N-terminal region of the calcium-binding proteolgycan testican 3, which has been reported to bind membrane-type MMPs including MMP-14 and to inhibit pro-MMP-2 activation in HEK293T cells when their respective cDNAs were co-transfected. These results led the authors to suggest that N-TES is an inhibitor of MMP-14 and MMP-16^47^.

Finally, we also produced and purified RECK fragment FRAG-1 resulting from the limited cleavage of RECKΔC by MMP-14 CD, which contained the C-terminal half of the full-length protein including KL1 through KL3 (Figs. 1 and 2G). MMP-14 CD was produced by *E. coli* BL21 (DE3) in inclusion bodies, purified by IEC under denaturing conditions, folded by dialysis, and finally purified by SEC by implementing a previous protocol^41^ (Fig. 2I,J).

### Proteolytic contamination and additional purification steps

**Figure 3:**
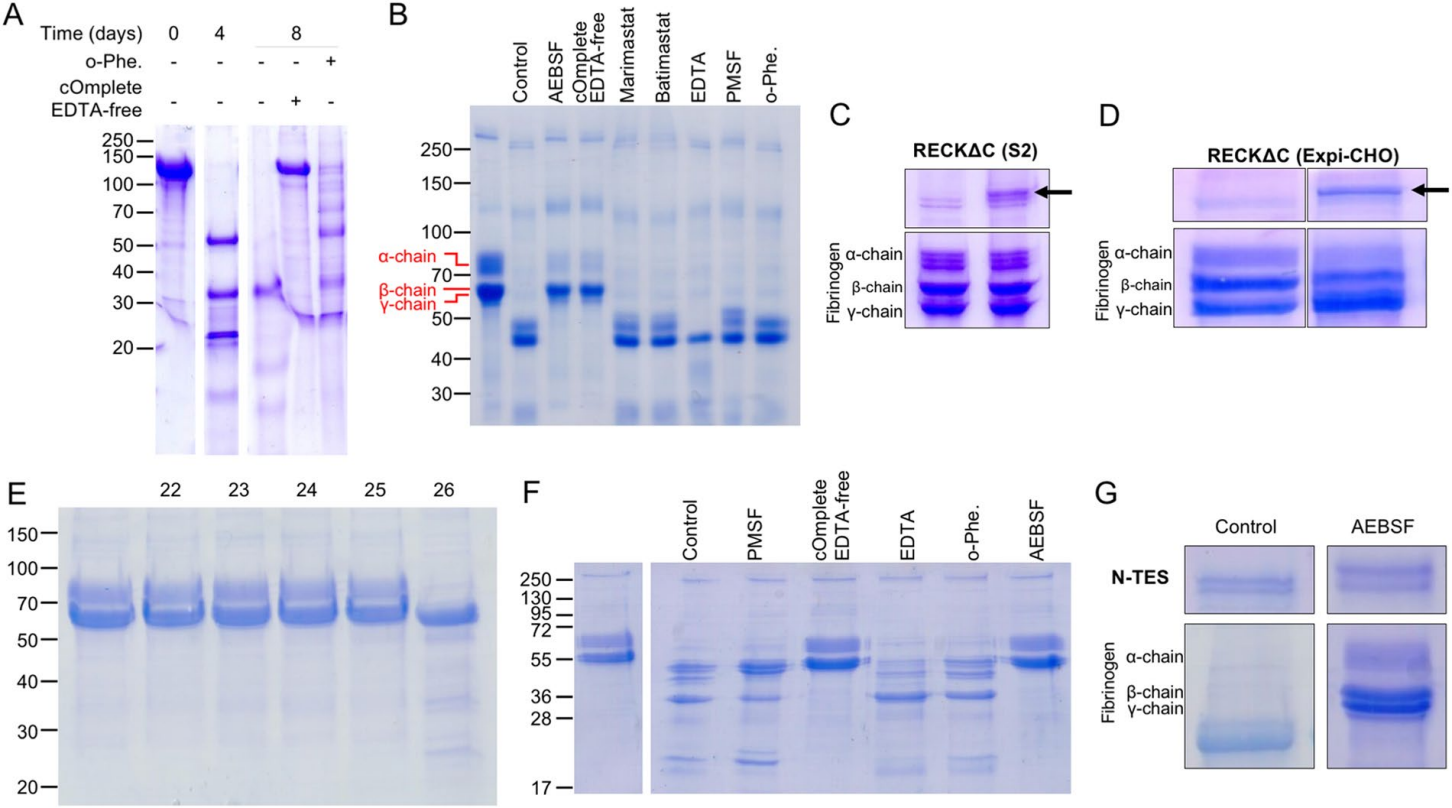
Functional assays. (**A**) Partially purified RECK∆C incubated for up to 8 days at 37 °C with or without o-phenanthroline (o-Phe.) or an inhibitor cocktail (cOmplete EDTA-free). (**B**) Degradation of fibrinogen. *Lane 1*, intact fibrinogen; *lane 2*, *control*, fibrinogen incubated with partially purified RECKΔC for two days shows degradation; *lanes 3–9*, same except for a previous one hour-incubation of RECKΔC with AEBSF, inhibitor cocktail, marimastat, batimastat, EDTA, PMSF or o-phenanthroline. Fibrinogen cleavage does not occur with similarly purified RECKΔC from (**C**) S2 cells or (**D**) Expi-CHO cells (both, *left lane*, fibrinogen alone; *right lane*, fibrinogen *plus* RECKΔC [black arrow]). (**E**) Incubation of fibrinogen (*lane 1*, *control*) with SEC fractions containing only FRAG-1 (22–25) show no degradation. However, the substrate is cleaved by a coeluting MMP-14 CD contamination (fraction 26). (**F**) Incubation of fibrinogen (*lane 1*) with partially purified N-TES without (*lane 2*, *control*) or with (*lanes 3–7*) inhibitors. (**G**) An N-TES preparation purified by SEC cleaved fibrinogen (*control)*. This cleavage was abolished with AEBSF. Figure panels with lanes/parts from different gels/blots show white separation lines. All original gels can be found in the supplementary materials.

Recombinant RECKΔC from Expi cells was initially purified by AC (Fig. 2E) and SEC. Despite rather high purity (>98%; Figs. 2E and 3A), it underwent cleavage over time, which was prevented by an inhibitor cocktail and partially slowed down by the general zinc chelator and MP inhibitor o-phenanthroline (Fig. 3A). However, this cleavage did not result in dissociation of RECKΔC in the short term, according to SEC. The cleavage products caused by this impurity (Fig. 3A, *lane 2*) were subjected to N-terminal Edman degradation, which revealed that a ~50-kDa fragment, dubbed FRAG-1, resulted from cleavage before G^484^ and thus comprised RECK domains KL1-KL2-KL3. As partially purified RECKΔC had initially shown slight inhibition of MMP-14 CD (data not shown), we speculated that RECKΔC cleavage might be necessary to yield a species with MMP inhibitory activity. Thus, we included FRAG-1 in subsequent inhibition assays (see below).

To investigate the nature of the proteolytic impurity, we incubated initially purified RECKΔC with the general peptidase substrate human plasma fibrinogen, and found it was cleaved (Fig. 3B). We assayed a series of peptidase inhibitors and found that AEBSF abolished cleavage (Fig. 3B). AEBSF is an irreversible small-molecule serine peptidase inhibitor that covalently modifies the catalytic serine of serine endopeptidases, thus blocking them. The same ablation was obtained with the inhibitor cocktail, which contained AEBSF, but not with PMSF or general MP inhibitors (Fig. 3B). This motivated us to include an extra step in the purification protocol of RECKΔC from Expi cells consisting of incubation with AEBSF and final polishing by several cycles of SEC. This protocol yielded RECKΔC of highest purity, incapable of fibrinogen or RECKΔC degradation, for subsequent inhibitory studies. Interestingly, protein produced from S2 or Expi-CHO cells did not show this contaminant and fibrinogen remained intact upon incubation with these RECKΔC species (Fig. 3C,D). Finally, FRAG-1 obtained from highly pure RECKΔC through treatment with MMP-14 CD did not contain the proteolytic contaminant of partially purified RECKΔC but some traces of the MP, which could be separated in SEC (Fig. 3E).

To assess whether the AEBSF-sensitive contaminant was a specific feature of the overexpression of RECKΔC, we studied protein N-TES obtained with the same expression system (Fig. 2H) and observed similar peptidolytic activity against fibrinogen that was abolished with AEBSF or the inhibitor cocktail (Fig. 3F,G). In contrast, two other proteins obtained with the same system did not contain this contaminant (data not shown).

### Inhibition studies of RECK and N-TES constructs

**Figure 4:**
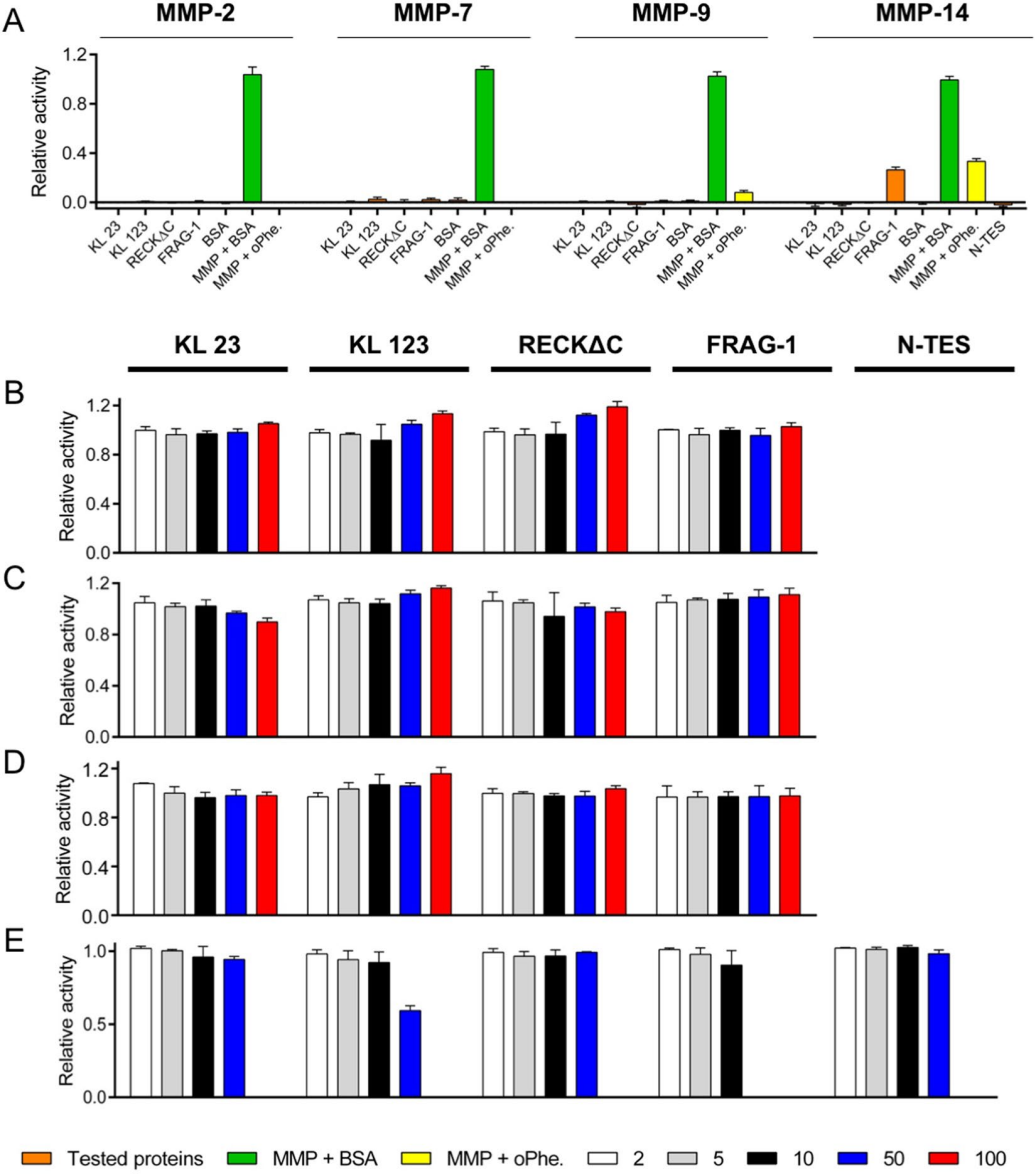
Activity and inhibition assays. (**A**) Hydrolytic activity of pure RECK constructs, N-TES and BSA (tested proteins, orange bars) relative to that of the respective MMPs (MMP-2, -7, -9 and -14) with BSA (100%; positive control, green bar) or o-phenantroline (negative control, yellow bar). Only FRAG-1 evinces slight residual activity against the fluorogenic substrate FS-6, possibly due to a contamination with MMP-14 CD used for its generation (see Fig. 2G). (**B**) Residual activity of MMP-2 in the presence of KL23, KL123, RECKΔC or FRAG-1 at various molar rations (1:2, 1:5, 1:10, 1:50 and 1:100; white, gray, black blue and red bars) after 1 h incubation relative to the activity shown in (A; green bar). (**C**) and (**D**), same as (**A**) for MMP-7 and MMP-9. (**E**) Same as (**A**) for MMP-14 but further including N-TES as candidate inhibitor up to 1:50 molar ratio. Only 50-fold excess of KL123 shows a slight inhibitory effect on MMP-14 CD activity. All assays of panels A-E were performed in triplicate.

**Figure 5:**
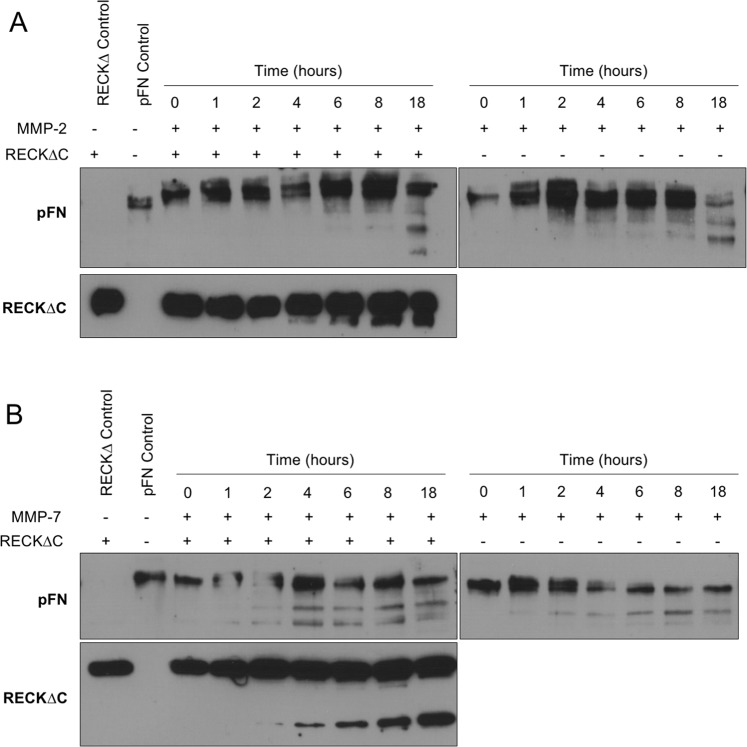
MMP-2 and MMP-7 activity assays against plasma fibronectin. (**A**) Western-blot analysis of the MMP-2 (40 nM) activity in front of plasma fibronectin (pFN; 4 nM) in the presence or absence of RECKΔC (400 nM) over time (0–18 h). The peptidase eventually cleaves the substrate irrespective of the presence of RECKΔC. (**B**) Same as (A) for MMP-7. In addition, MMP-7 cleaves RECKΔC over time. Figure panels with lanes/parts from different gels/blots show white separation lines. All original gels can be found in the supplementary materials.

Highly pure RECK variants RECK∆C, KL123, KL23 and FRAG-1, as well as N-TES, were tested for their inhibitory capacity against MMP-2, MMP-7, MMP-9 and MMP-14 activity with peptide and protein substrates up to 100-fold molar excess of the tester proteins (Figs. 4 and 5). Fluorescein-conjugated gelatine was used for assays with previously activated MMP-2 (Fig. 4B), MMP-7 (Fig. 4C) and MMP-9 (Fig. 4D), and fluorogenic peptide FS-6 was employed for MMP14 CD (Fig. 4E). In addition, inhibition of the activity of MMP-2 (Fig. 5A) and MMP-7 (Fig. 5B) against plasma fibronectin by RECKΔC at tenfold molar excess was assayed by Western blot analysis. Moreover, inhibition of the activity of MMP-14 CD against a fluorogenic peptide substrate by N-TES was likewise analysed (Fig. 4E). As expected, none of the RECK constructs, N-TES or BSA, which was used as a negative control, alone showed relevant peptidolytic activity and o-phenanthroline inhibited the MMPs (Fig. 4A). In addition, BSA at the highest tester concentration (1:100 molar excess) had no influence on MMP activity (Fig. 4A). Notably, the experiments revealed that none of the RECK constructs or N-TES showed any significant inhibition of MMPs activity.

### Conclusions

Since its discovery in the 1980s, protein RECK has been found to have pleiotropic roles, for example in embryogenesis and as a tumour suppressor. Among other functions, it has been hailed as an MMP inhibitor. Here, we produced two RECK variants through the mammalian Expi and Expi-CHO systems and through the insect S2 system. We could not detect any significant inhibition with either construct produced with Expi when we assayed MMPs that have been previously reported to be targeted, neither with peptide nor protein substrates. Instead, we found that even quite pure samples of RECKΔC showed proteolytic activity resulting from a contamination by a probable serine peptidase, which could be removed by treatment with AEBSF and additional SEC. This activity was missing when the protein was produced in Expi-CHO or S2 cells. We further established a production and purification protocol for N-TES, which has also been postulated to be an MMP inhibitor. As in the case of RECK, we detected the contaminating peptidase but no inhibitory activity in front of the target MMP-14. In contrast, similar expression systems for two other unrelated proteins did not produce the contaminant in the supernatant. Finally, we made two shorter constructs of RECK spanning KL1-KL2-KL3 and KL2-KL3, respectively, through an *E. coli* system, which also lacked the contaminant. These proteins did not show any inhibitory effect on MMPs either.

These results are consistent with marginal notes in a recent article describing very sensitive gelatine zymography, which revealed gelatinolytic activity associated with recombinant RECKΔC preparations^23^. These corresponded to co-purified peptidases of ~19 and ~28 kDa, which necessarily must have perturbed previously published kinetic assays with partially purified RECKΔC from supernatants of transfected 293 F and 293 T cells, as well as construct K23 from 293 T cells, for which inhibitory activity on MMPs had been reported^13,15–17^. Moreover, the inhibitory activity of RECK on MMPs was requalified as “weak” recently^36^.

Taken together, all these findings suggest that RECK, and probably N-TES, are not direct inhibitors of MMP catalytic activity. Instead, RECK may still regulate MMPs *in vivo* at a different level, e.g. through downregulation of MMP transcription, translation or secretion, or by binding and sequestering them, thus preventing them from carrying out their extracellular peptidolytic function.

Finally, we recommend checking for unexpected proteolytic activity associated with recombinant proteins of interest when employing transfected Expi293F or other HEK293-derived cells as protein expression systems. This activity can be removed through irreversible serine peptidase inhibitors and is absent from systems based on Chinese hamster ovary or fruit-fly S2 cells.

## Supplementary information

Supplementary Information.
