# Supplementary material for: Analysis of the inhibiting activity of reversion-inducing cysteine-rich protein with Kazal motifs (RECK) on matrix metalloproteinases
Source: Sci Rep. 2020 Apr 14;10:6317. doi: 10.1038/s41598-020-63338-4 (PMC7156630; doi:10.1038/s41598-020-63338-4)

Analysis of the inhibiting activity of reversion-inducing cysteine-rich protein with Kazal motifs (RECK) on matrix metalloproteinases

Soraia R. Mendes, Laura del Amo-Maestro, Laura Marino-Puertas, Iñaki de Diego, Theodoros Goulas & F. Xavier Gomis-Rüth

Original Gels for Figures

Figure 2A

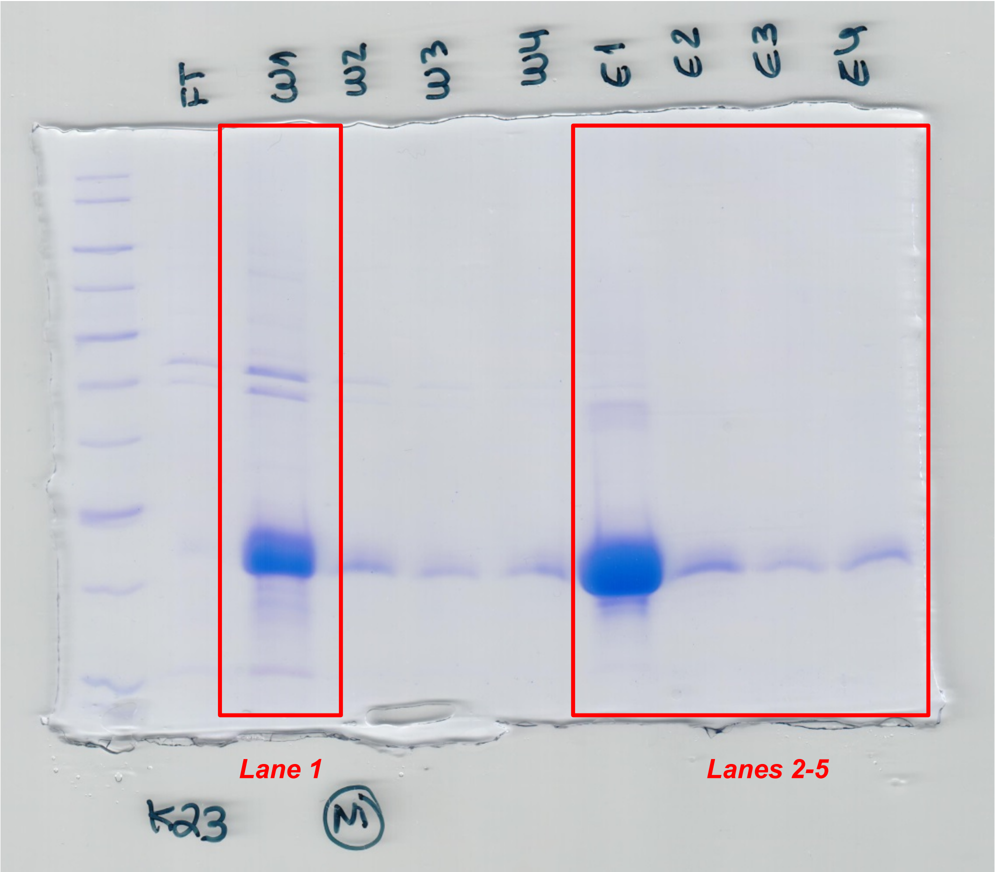

Figure 2B

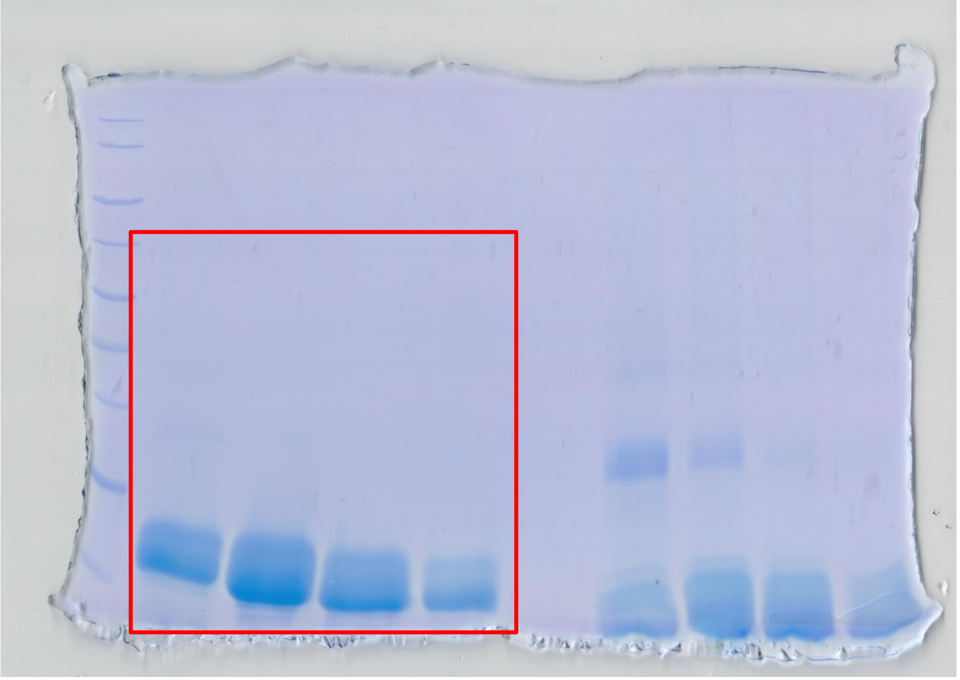

Figure 2C

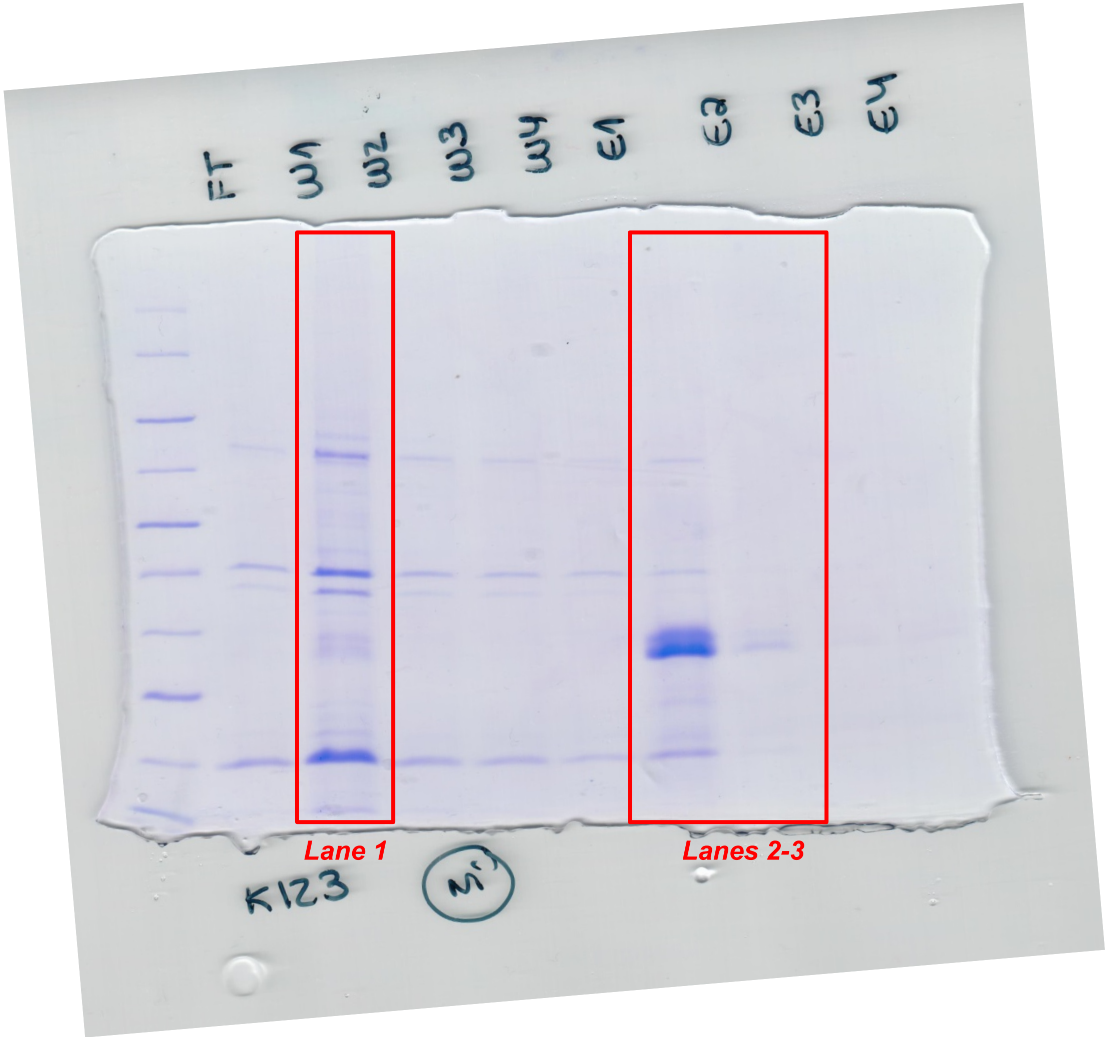

Figure 2D

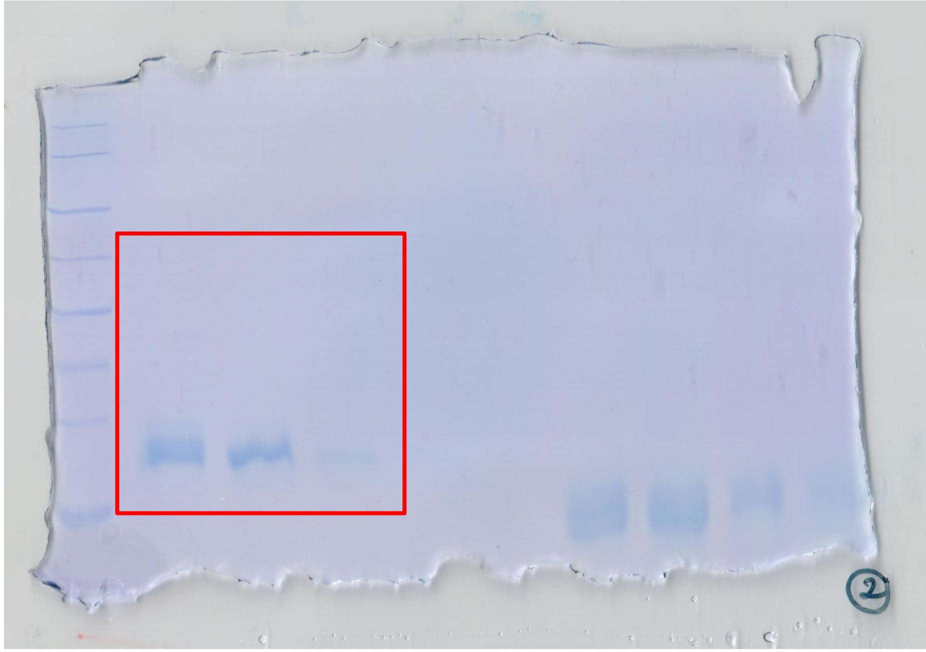

Figure 2E

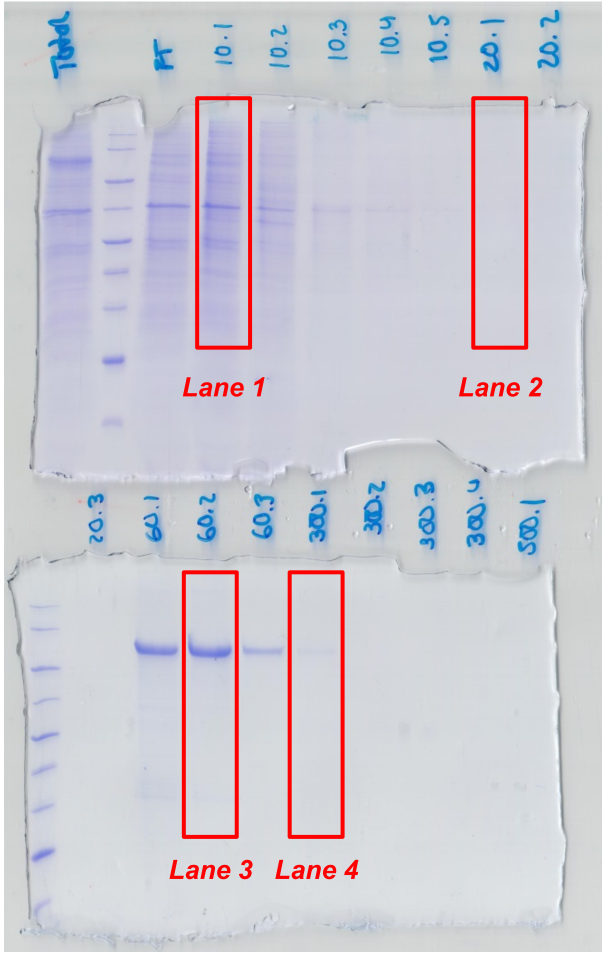

Total
FT
10.1
10.2
10.3
10.4
10.5
20.1
20.2
Lane 1
Lane 2
20.3
60.1
60.2
60.3
300.1
300.2
300.3
300.4
500.1
Lane 3
Lane 4

Figure 2G

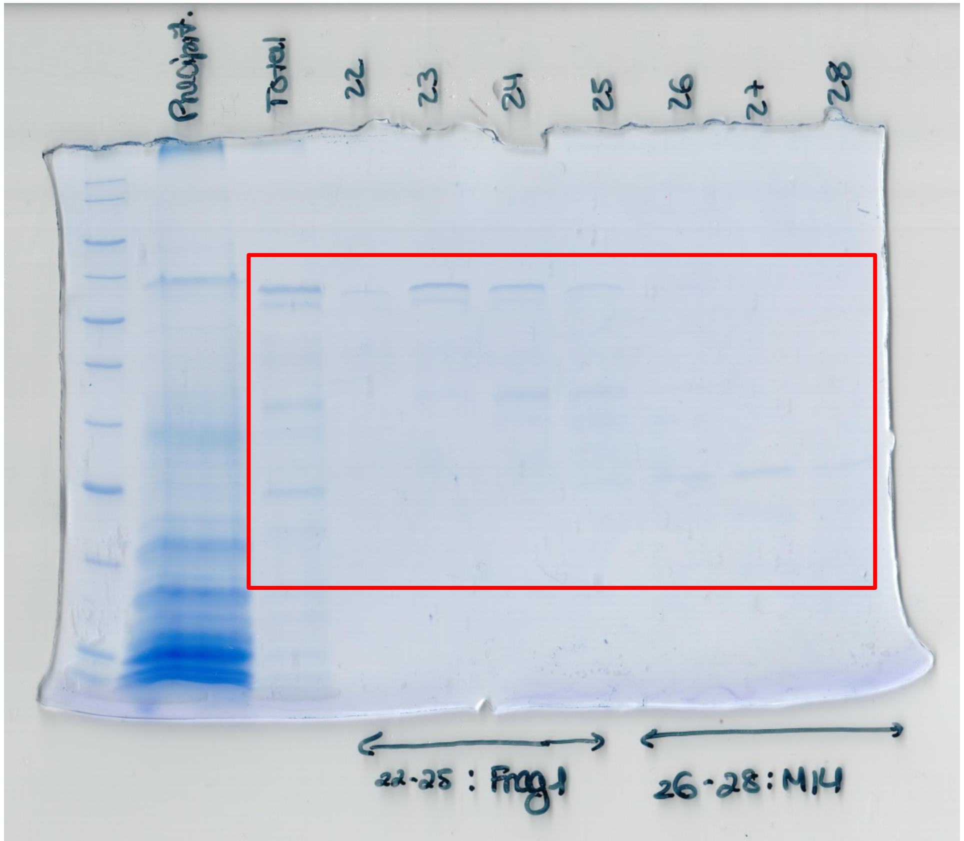

Figure 2H

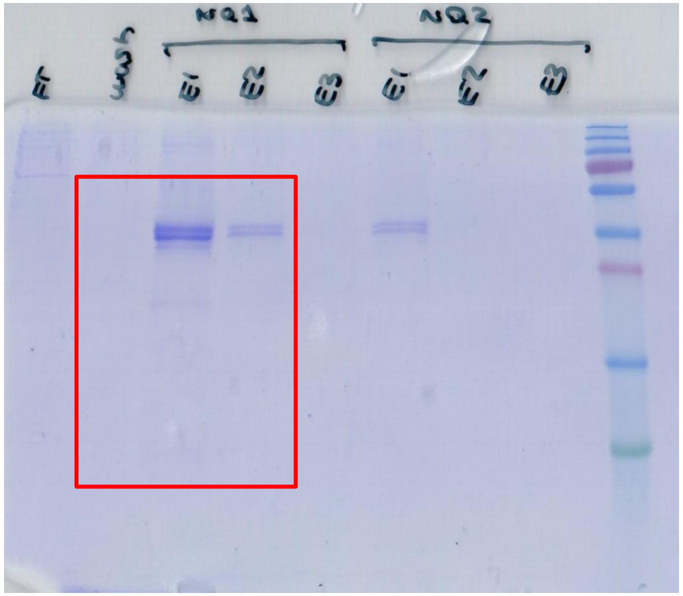

Figure 2I

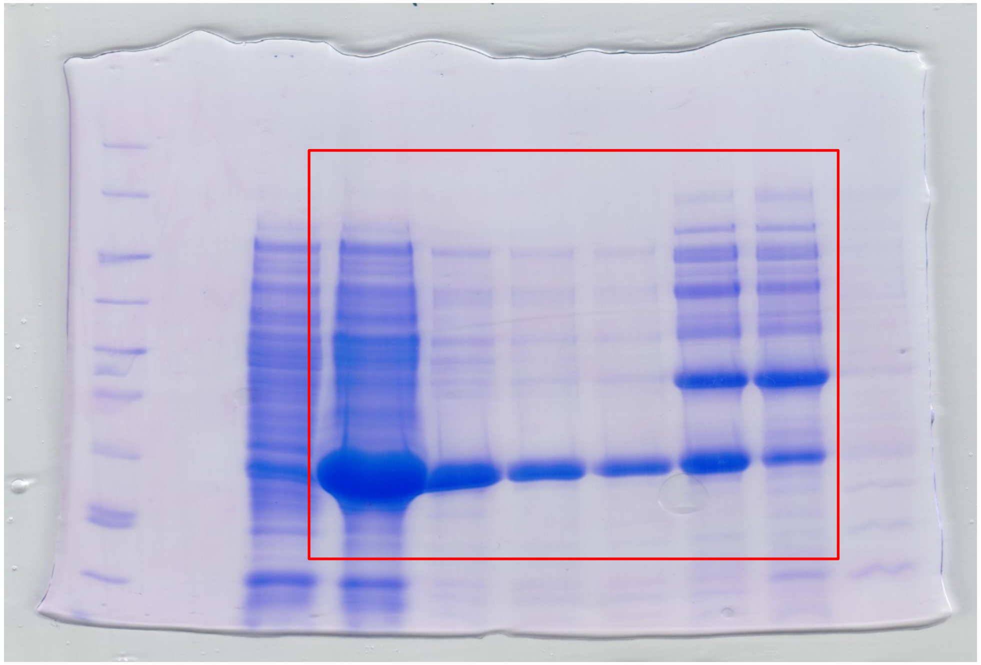

Figure 2J

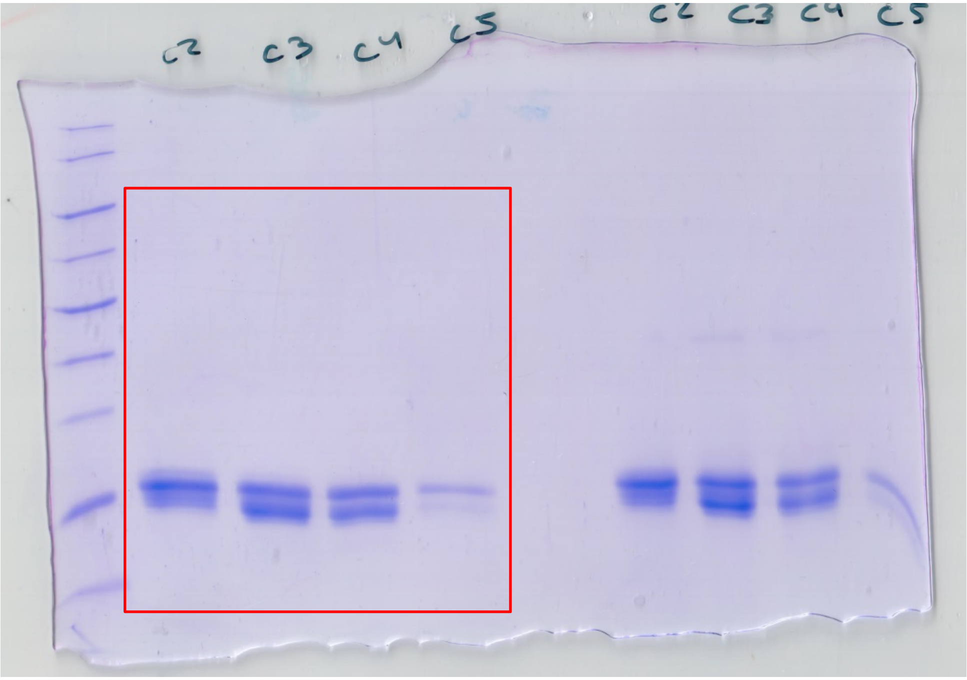

Figure 3A

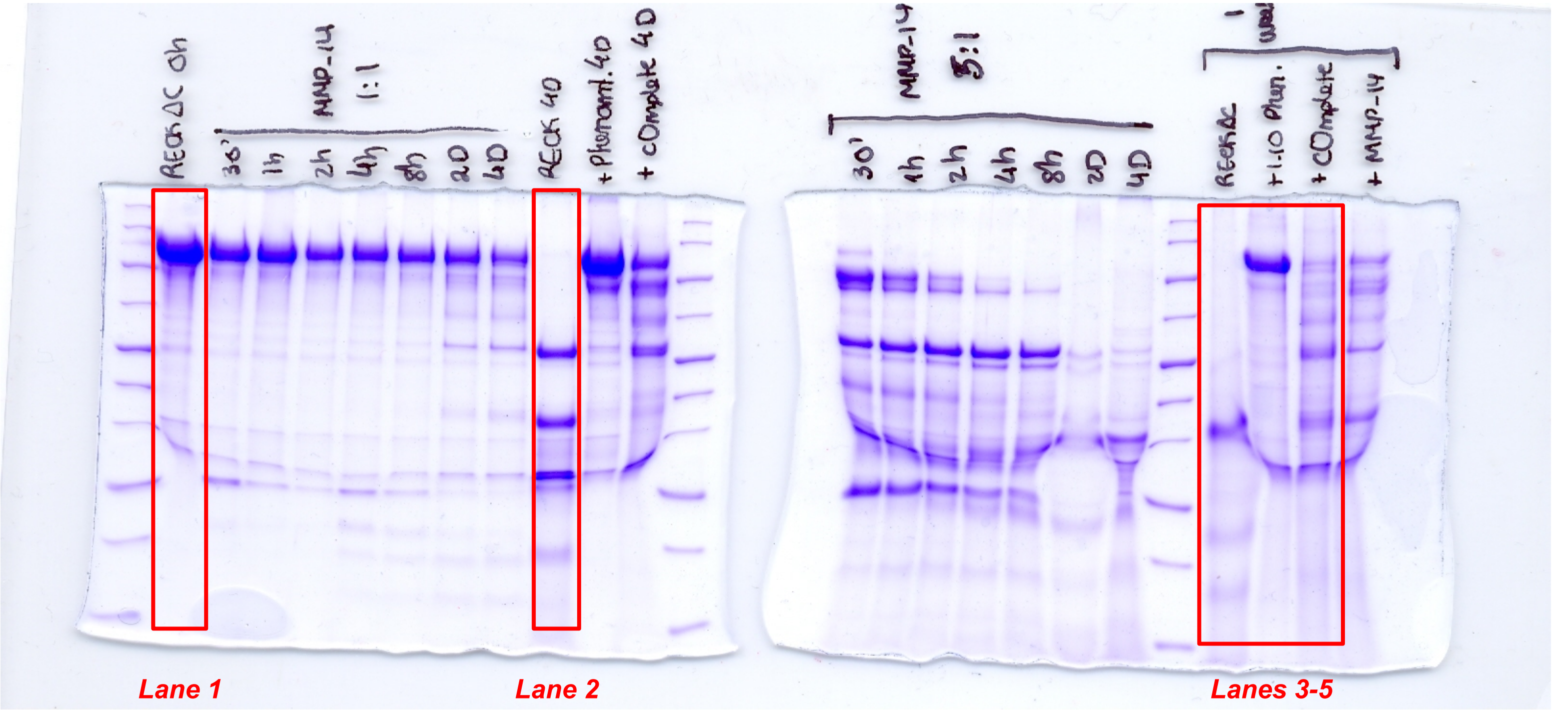

Figure 3B

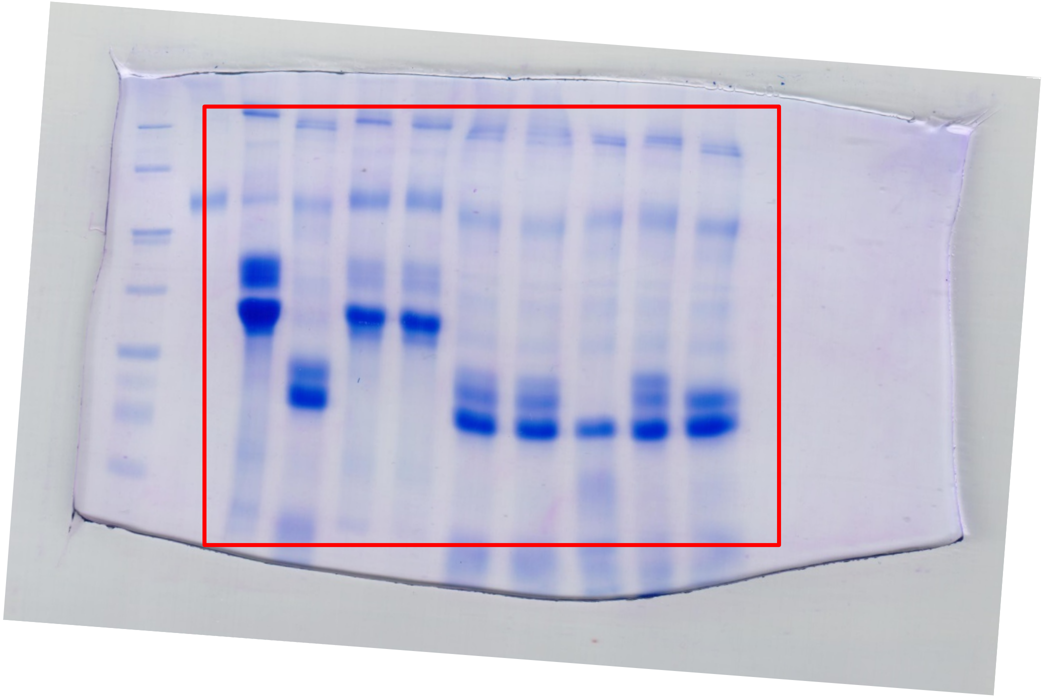

Figure 3C

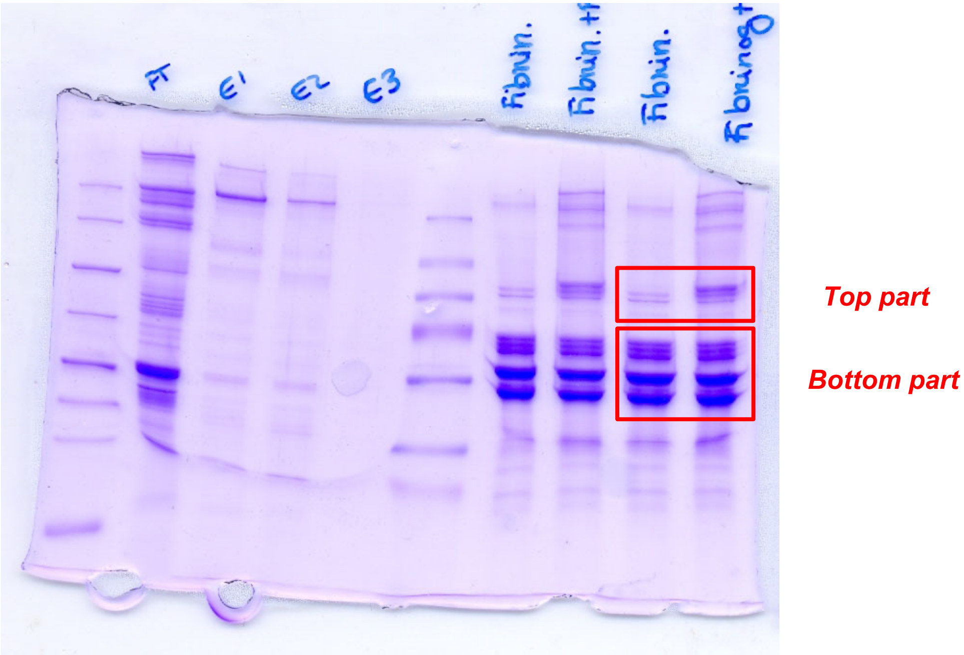

Figure 3D

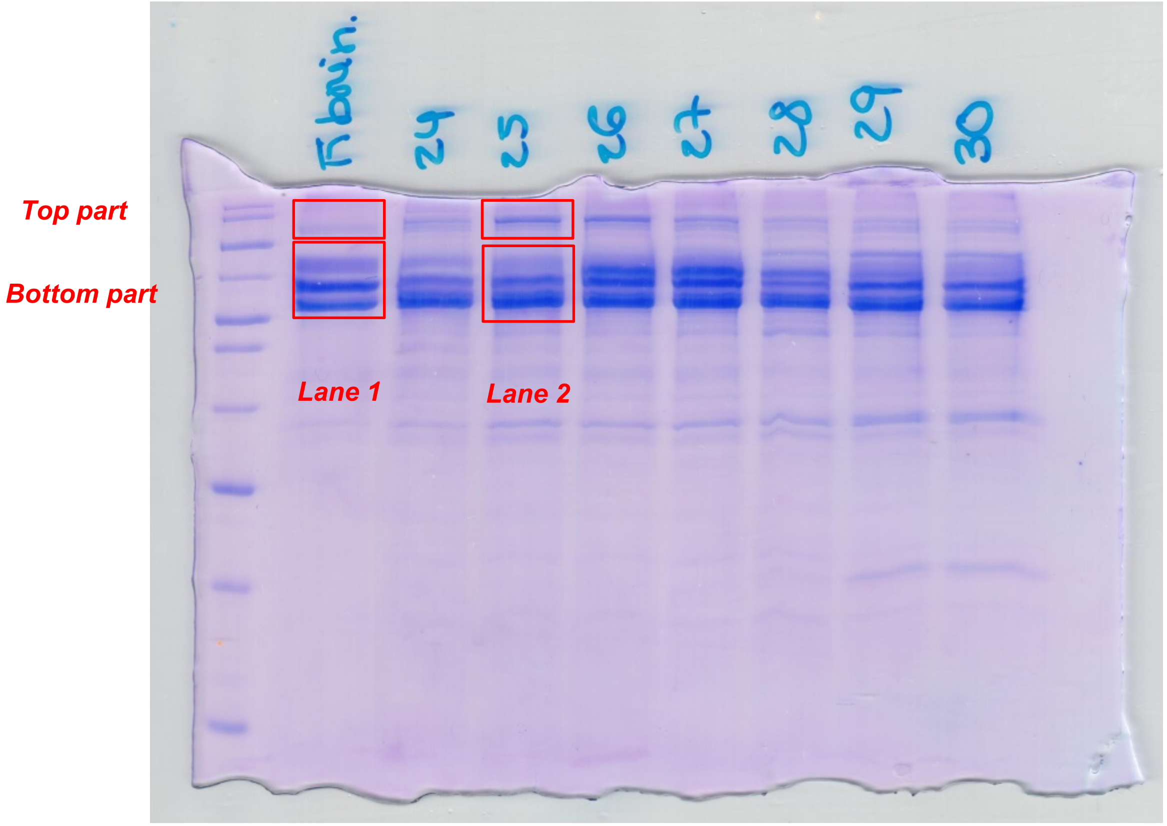

Figure 3E

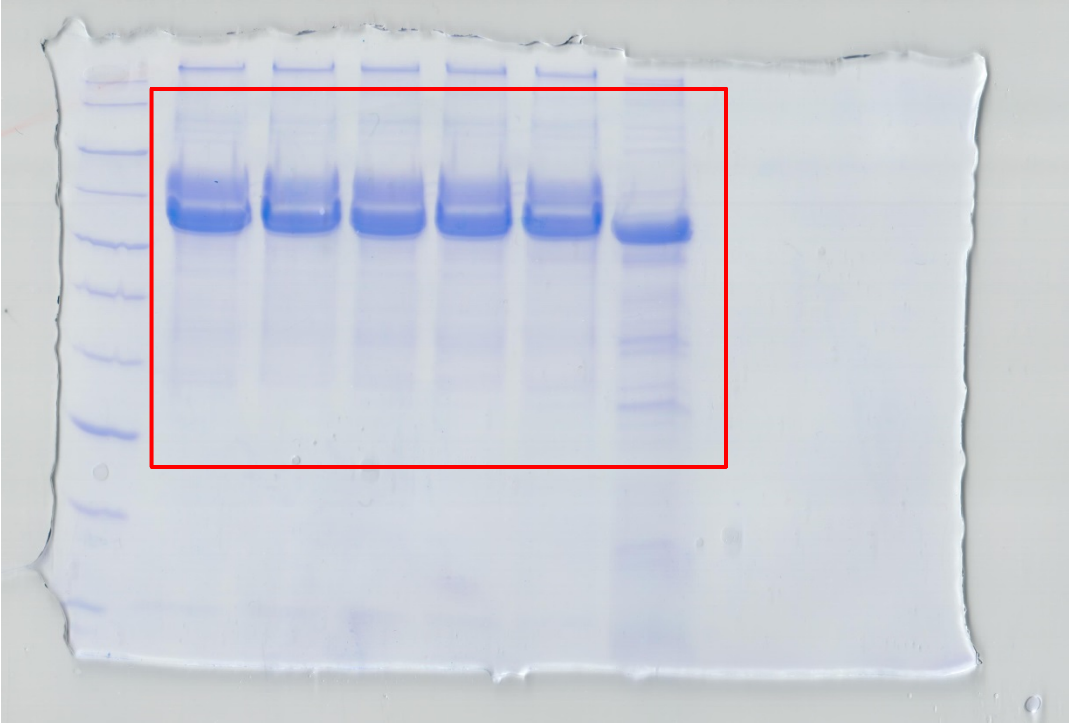

Figure 3F

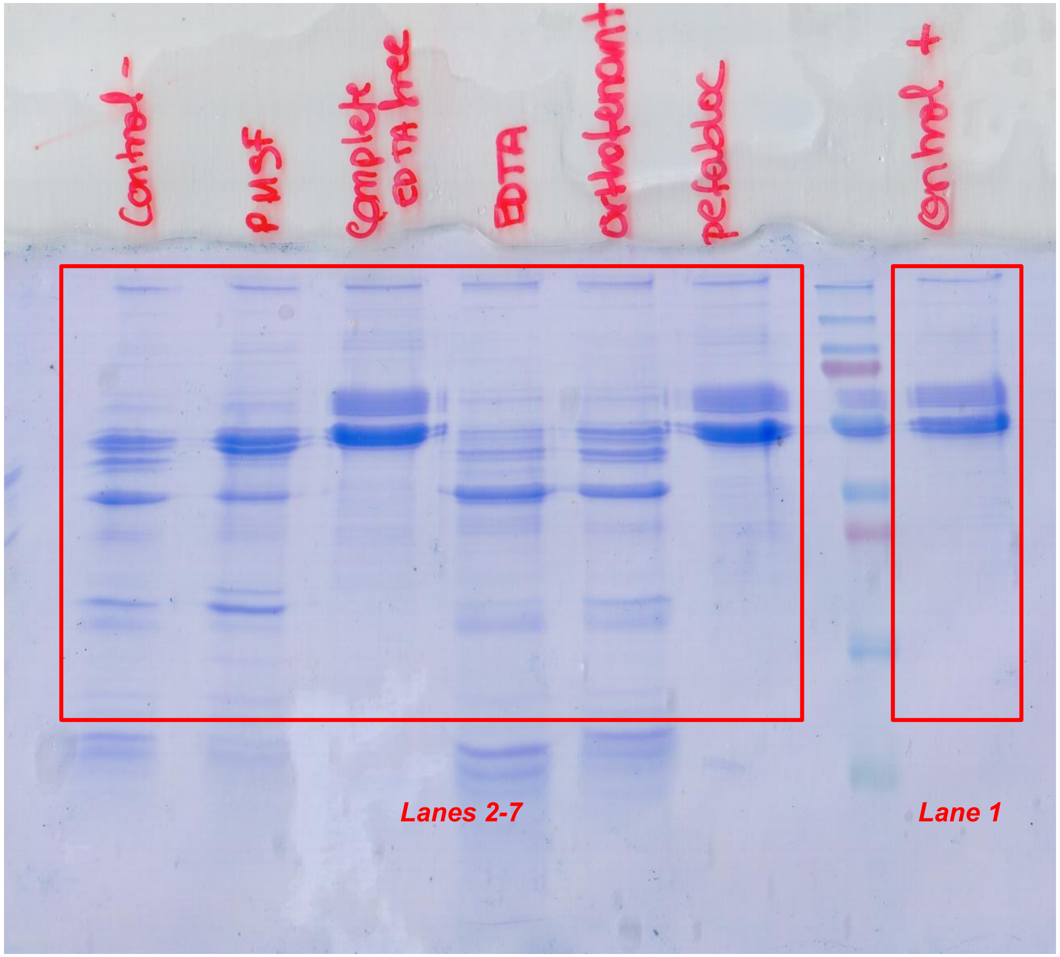
Control -
PMSF
Complete EDTA free
EDTA
pefabloc
Control +
Lanes 2-7
Lane 1

Figure 3G

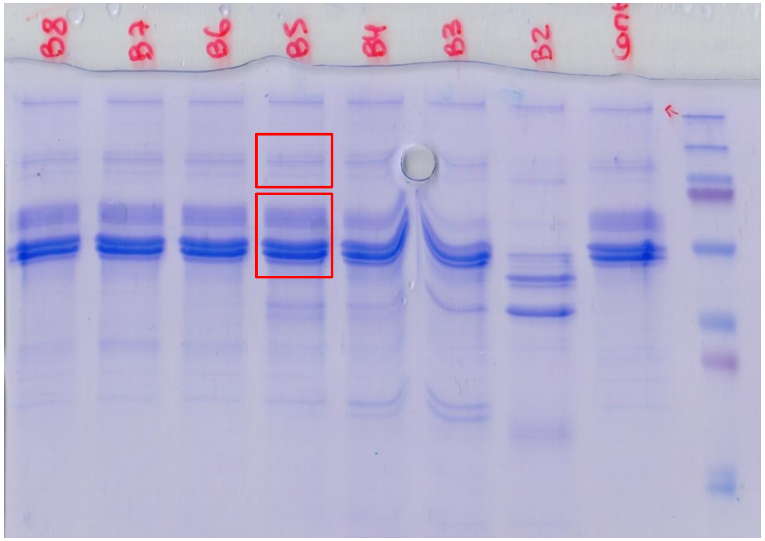

Lane 2

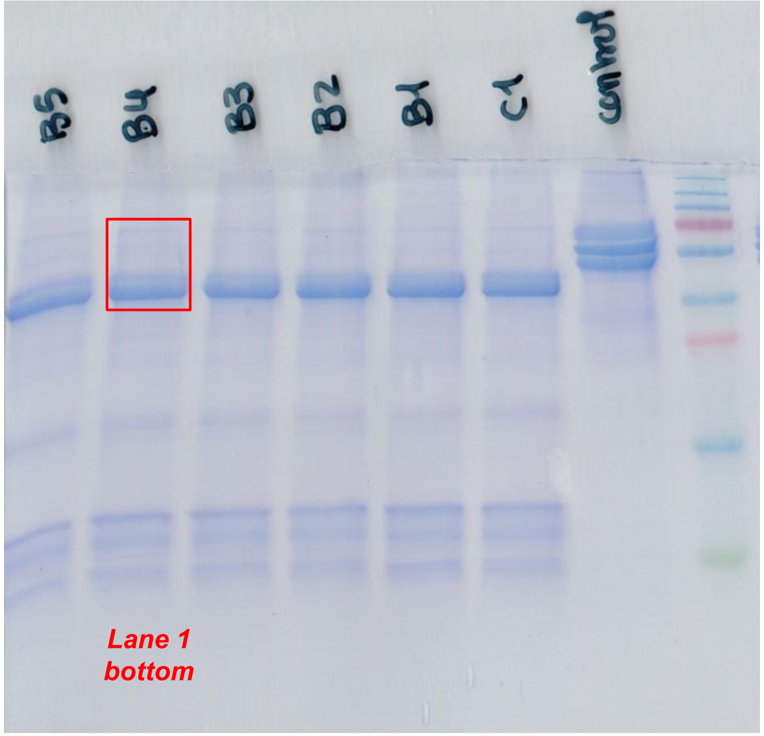

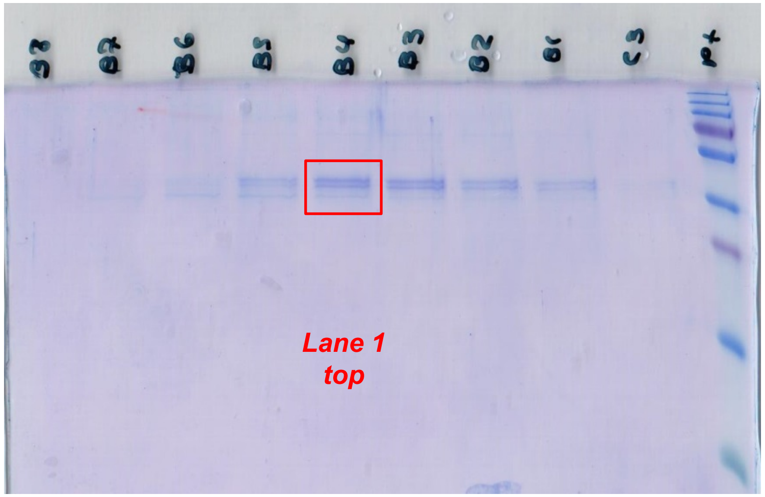

Figure 5A

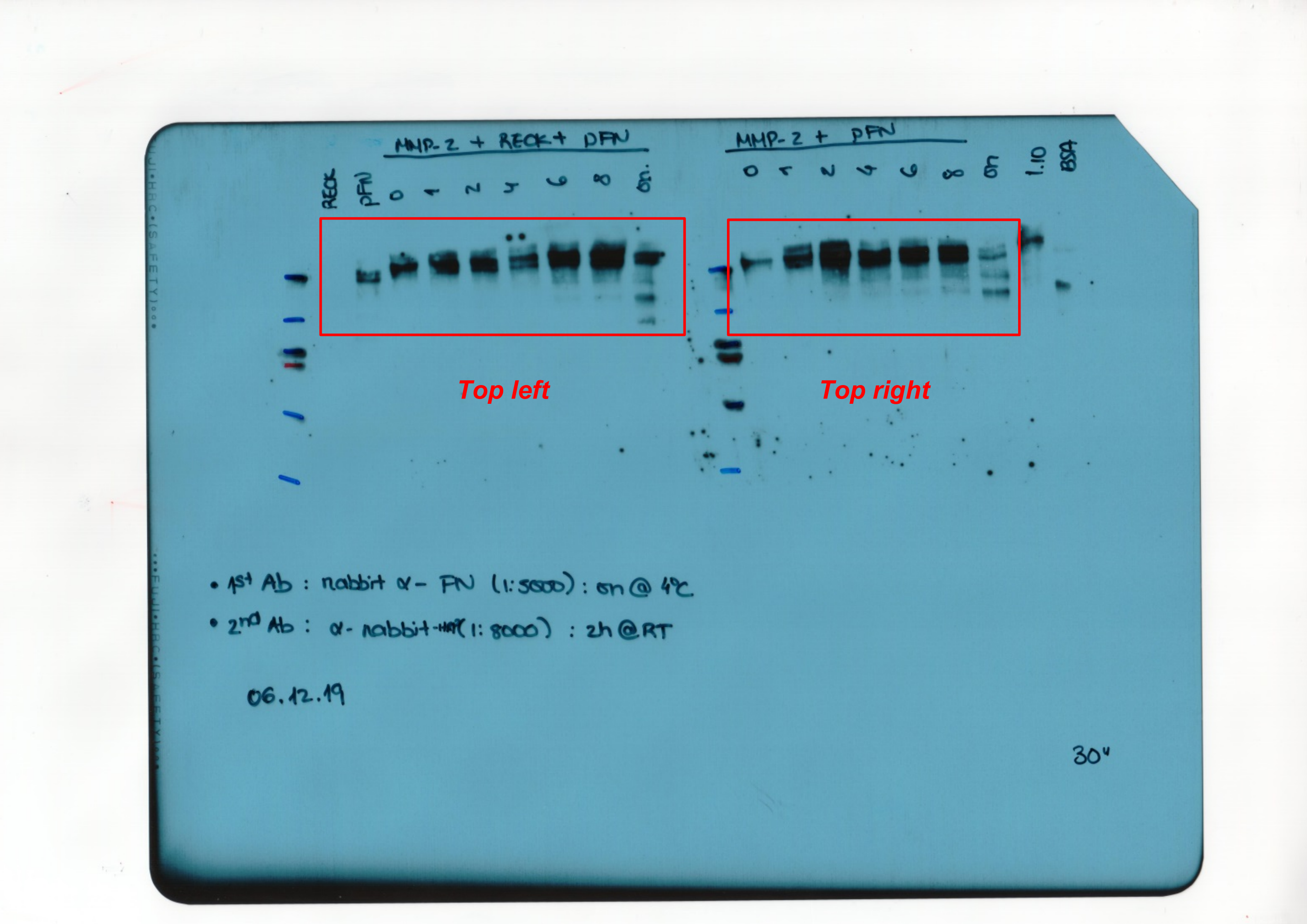

MMP-2 + RECK + PFN
MMP-2 + PFN
Top left
Top right
1st Ab : rabbit α- FN (1:5000) : on @ 4°C
2nd Ab : α- rabbit-HRP (1: 8000) : 2h @RT
06.12.19
30"

Figure 5A

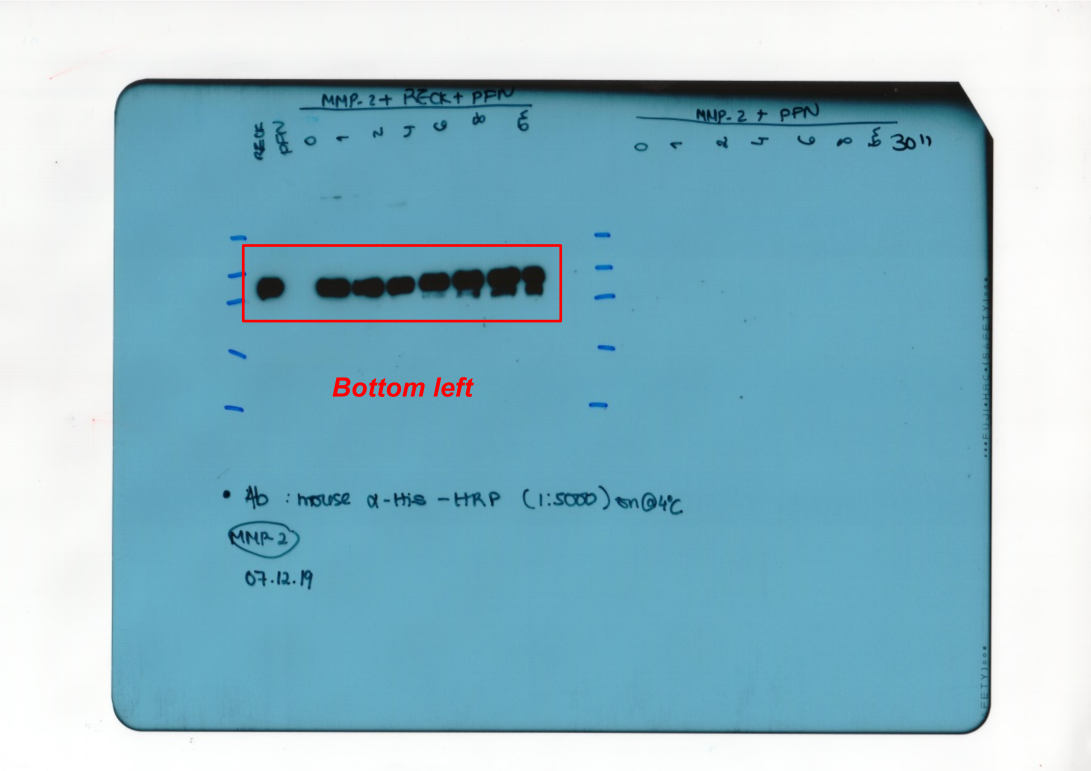

Figure 5B

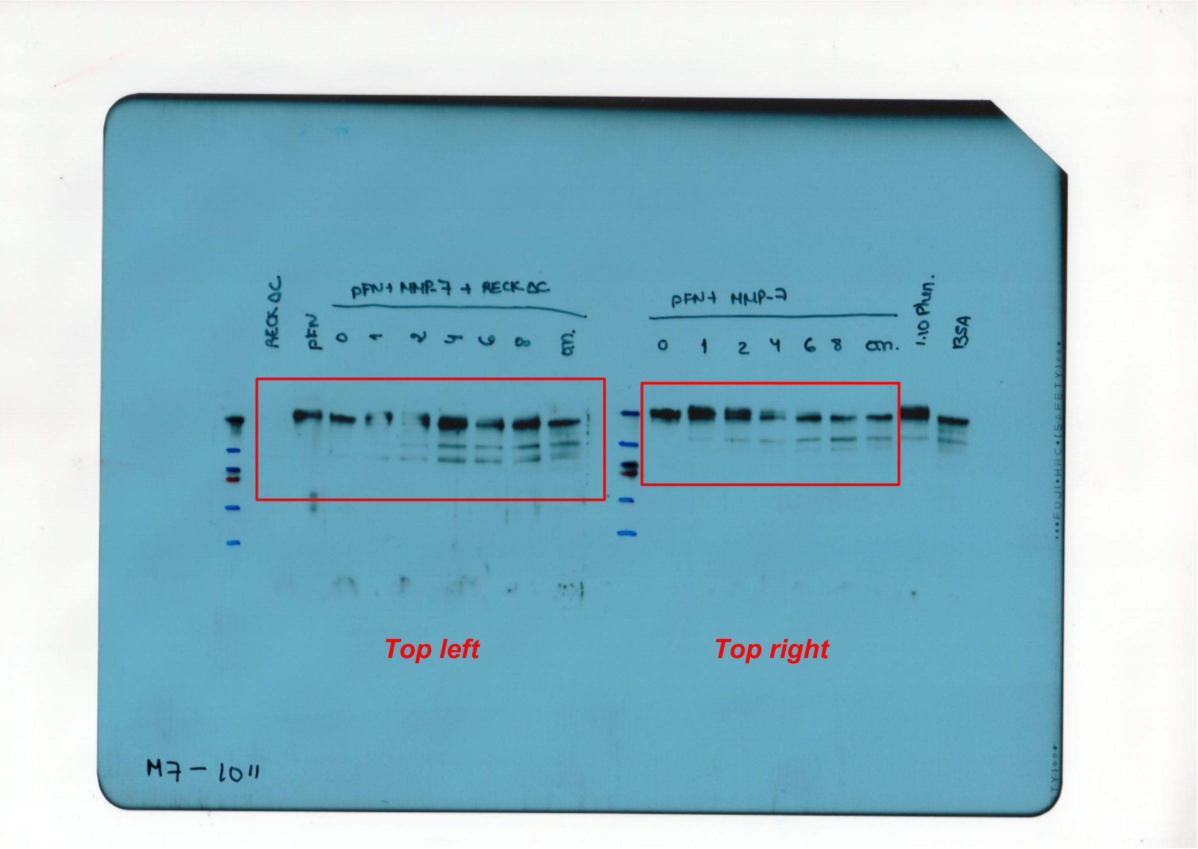

RECK ΔC
pFN
pFN+ MMP7 + RECK ΔC
pFN+ MMP-7
1.10 Phen.
BSA
0 1 2 4 6 8 on.
Top left
Top right
M7 – 10"

Figure 5B

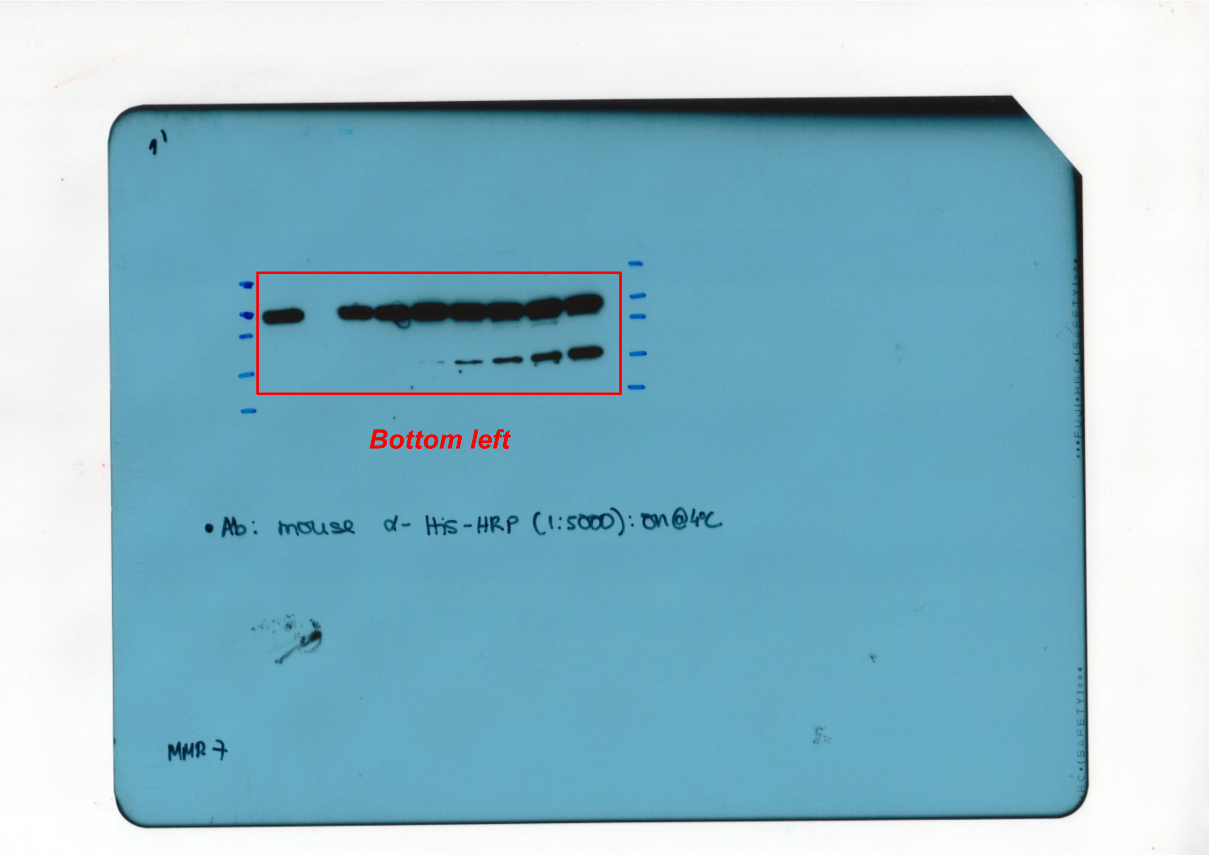

Supplement: Supplementary file 1 — Supplementary Information. [file 41598_2020_63338_MOESM1_ESM.pdf]
